# Genetic dissection of growth, wood basic density and gene expression in interspecific backcrosses of *Eucalyptus grandis* and *E. urophylla*

**DOI:** 10.1186/1471-2156-13-60

**Published:** 2012-07-20

**Authors:** Anand Raj Kumar Kullan, Maria M van Dyk, Charles A Hefer, Nicoletta Jones, Arnulf Kanzler, Alexander A Myburg

**Affiliations:** 1 Department of Genetics, Forestry and Agricultural Biotechnology Institute (FABI), University of Pretoria, Pretoria, 0002, South Africa; 2 Sappi Forests Research, Shaw Research Centre, PO Box 473, Howick, 3290, South Africa; 3 Bioinformatics and Computational Biology Unit, University of Pretoria, Pretoria, 0002, South Africa

## Abstract

**Background:**

F_1_ hybrid clones of *Eucalyptus grandis* and *E. urophylla* are widely grown for pulp and paper production in tropical and subtropical regions. Volume growth and wood quality are priority objectives in *Eucalyptus* tree improvement. The molecular basis of quantitative variation and trait expression in eucalypt hybrids, however, remains largely unknown. The recent availability of a draft genome sequence (http://www.phytozome.net) and genome-wide genotyping platforms, combined with high levels of genetic variation and high linkage disequilibrium in hybrid crosses, greatly facilitate the detection of quantitative trait loci (QTLs) as well as underlying candidate genes for growth and wood property traits. In this study, we used Diversity Arrays Technology markers to assess the genetic architecture of volume growth (diameter at breast height, DBH) and wood basic density in four-year-old progeny of an interspecific backcross pedigree of *E. grandis* and *E. urophylla*. In addition, we used Illumina RNA-Seq expression profiling in the *E. urophylla* backcross family to identify cis- and trans-acting polymorphisms (eQTLs) affecting transcript abundance of genes underlying QTLs for wood basic density.

**Results:**

A total of five QTLs for DBH and 12 for wood basic density were identified in the two backcross families. Individual QTLs for DBH and wood basic density explained 3.1 to 12.2% of phenotypic variation. Candidate genes underlying QTLs for wood basic density on linkage groups 8 and 9 were found to share trans-acting eQTLs located on linkage groups 4 and 10, which in turn coincided with QTLs for wood basic density suggesting that these QTLs represent segregating components of an underlying transcriptional network.

**Conclusion:**

This is the first demonstration of the use of next-generation expression profiling to quantify transcript abundance in a segregating tree population and identify candidate genes potentially affecting wood property variation. The QTLs identified in this study provide a resource for identifying candidate genes and developing molecular markers for marker-assisted breeding of volume growth and wood basic density. Our results suggest that integrated analysis of transcript and trait variation in eucalypt hybrids can be used to dissect the molecular basis of quantitative variation in wood property traits.

## Background

*Eucalyptus* tree species and hybrids are now the most widely planted hardwoods in tropical, subtropical and temperate regions, primarily due to their fast growth, short rotation, high productivity, adaptability to a broad range of environments and suitability for pulp and paper production [1,2]. Eucalypt plantations currently occupy more than 20 million hectares in over 50 countries. India (3.9 million hectares), Brazil (3.7 million hectares) and China (2.6 million hectares) are the leading eucalypt growers in the world (http://www.git-forestry.com). Commonly planted eucalypts are mainly from the subgenus *Symphyomyrtus,* sections *Latoangulatae* (*E. grandisE. urophylla*), *Maidenaria* (*E. globulus, E. nitens*) and *Exsertaria* (*E. camaldulensis, E. tereticornis*) and include hybrids of some of these species [1].

*Eucalyptus* breeding programs mainly focus on primary growth traits, such as height and volume growth (diameter at breast height, DBH) and wood quality traits including physical properties such as wood density and chemical properties such as cellulose and lignin content. Wood basic density (oven dry mass per green volume) is an important trait for kraft pulp production [3] as it affects specific wood consumption. The genetics of growth and wood property traits has been studied in *Eucalyptus*[3-5] and the heritability of growth traits is generally reported to be lower than that of wood quality traits [6,7] due to the large numbers of genes involved and environmental effects impacting on growth. The efficiency of selection for these traits could be enhanced by molecular breeding approaches enabled by the availability of high-throughput, genome-wide genotyping technologies [8,9] and the recent completion of a draft reference genome sequence for *Eucalyptus* (*E. grandis* V1.0, JGI, http://www.phytozome.net).

The identification of genetic factors underlying quantitative variation for growth and wood properties is important for tree breeding as well as gene discovery efforts. QTL analysis in structured pedigrees allows the identification of genomic regions harbouring candidate genes and trait-linked molecular markers [10]. Over the past decades, with the advancement of DNA marker technologies and genome-wide linkage mapping, major efforts have been dedicated to the genetic dissection of growth and wood quality traits in eucalypts [11-18]. The number of QTLs detected for growth traits in *Eucalyptus* has generally been lower (1–3) than that detected for wood quality traits (3–7), which may reflect the lower heritability associated with growth traits and the lack of statistical power to detect many small-effect growth QTLs segregating in tree breeding pedigrees. In addition, the number of QTLs have, most likely, been underestimated and the magnitude of QTL effects overestimated due to the relatively small progeny sample sizes used for QTL detection (100 to 200 individuals) [19]. QTL intervals normally span 10–30 cM regions, which may contain thousands of genes in *Eucalyptus*[20,21]. The identification of positional candidate genes by linkage mapping in trees therefore remains a difficult task.

To partly overcome the limited resolution of QTL analysis, genetical genomics approaches have been used to narrow down the list of candidate genes in QTL intervals in several plant species including eucalypts [13,22-25]. In these approaches, the transcript levels of individual genes, generally obtained from microarray analyses, are treated as separate quantitative phenotypes and chromosomal regions affecting transcript variation (i.e. expression QTLs or eQTLs) are identified using conventional QTL detection methods. Comparison of trait QTL and eQTL positions enables the identification of candidate genes, which may potentially affect phenotypic traits through variation in transcript abundance and downstream effects on protein abundance and activity [26]. eQTL analyses performed in *Arabidopsis*[22,27-29], maize [30], wheat [31] and *Populus*[32,33], have allowed elucidation of the genetic control of biochemical pathways as well as genetic correlations observed in populations. In *Eucalyptus,* Kirst *et al.*[13] identified QTLs for transcript levels (i.e., eQTLs) of lignin-associated genes, which co-localized with QTLs for growth, suggesting that the same genomic regions may affect lignin content and tree growth.

*Eucalyptus grandis* is extensively used for the production of pulp in subtropical regions because of its rapid growth, however the species suffers from fungal diseases in tropical regions [34]. *Eucalyptus urophylla*, a tropical eucalypt found in natural forests in Indonesia*,* is more disease tolerant than *E. grandis.* Interspecific hybrids of these two species combine the fast growth and successful vegetative propagation of *E. grandis* with the greater tolerance to fungal disease, excellent adaptability, higher wood density and greater coppicing capability of *E. urophylla*[35,36]. The identification of genetic factors that differentiate these two species and that may underlie hybrid superiority are therefore important for *Eucalyptus* breeding programs and for fundamental understanding of growth and development in hybrids. Currently in *Eucalyptus,* advanced generation hybrid breeding and gene pyramiding are hampered by long generation times, hybrid breakdown, inbreeding depression and limited knowledge of genetic factors controlling interspecific trait variation. Knowledge of gene expression (eQTL) and trait QTLs associated with the trait variation, their main effects and interactions with other QTLs will facilitate marker-assisted selection in *E. grandis* or *E. urophylla* parents (for F_1_ hybrid breeding), or in hybrid progeny (for advanced generation hybrid breeding).

The aim of this study was to dissect the molecular genetic basis of diameter at breast height (DBH) and wood basic density in an interspecific backcross pedigree of *E. grandis* x *E. urophylla*[37]. Herein we report QTLs for both traits and we demonstrate the potential use of expression QTL (eQTL) analysis for dissecting cis- and trans-acting components of genetic variation underlying major QTLs for wood basic density.

## Results

### Trait distribution and correlation

**Figure 1:**
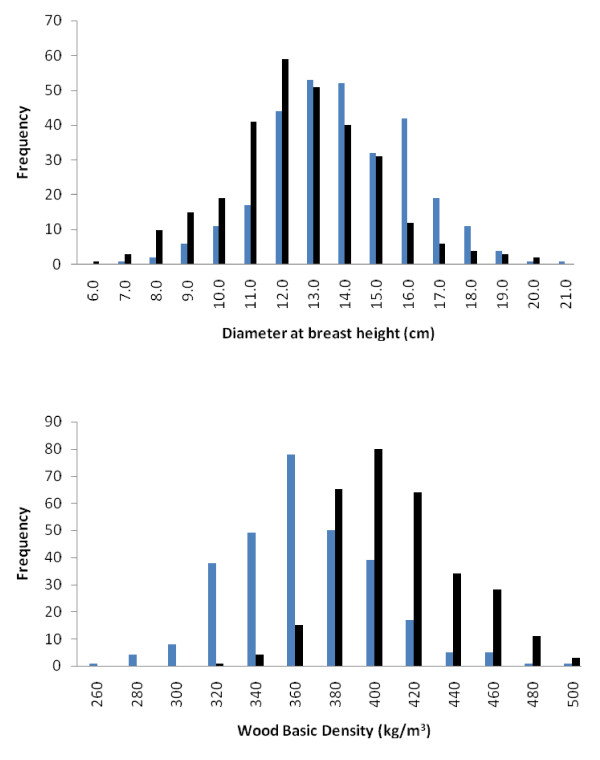
**Distribution of DBH (upper panel) and wood basic density (lower panel) trait values at four years of age in the*E. grandis*(blue) and*E. urophylla*(black) backcross families.** The *E. grandis* backcross family had slightly higher average DBH than the *E. urophylla* backcross family (15.4 vs. 13.0 cm, Table 1), while the *E. urophylla* backcross family had higher average wood basic density (410 vs. 305 kg/m^3^).

DBH and wood basic density (referred to as wood density hereafter) at four years showed wide quantitative variability and approximately normal frequency distribution in the two F_2_ backcross (BC) families (Figure 1, Table 1). The mean wood density observed in the *E. urophylla* BC family was higher than that observed for the *E. grandis* BC family (410 vs. 305 kg/m^3^, Table 1). DBH and wood density were not significantly correlated in either backcross family (*r =* 0.06 for *E. grandis* BC and −0.02 for *E. urophylla* BC).

**Table 1:** **Summary statistics of DBH and wood basic density measured at age four years in the*E. grandis*and*E. urophylla*backcross families**

**Statistics**	***E. grandis*BC family**		***E. urophylla*BC family**	
	**DBH (cm)**	**Density (kg/m**^**3**^**)**	**DBH (cm)**	**Density (kg/m**^**3**^**)**
Mean	15.4	305	13.0	410
SD	2.4	30	2.3	30
Min	7.7	258	6.8	320
Max	22.5	495	20.3	508
N	286	286	308	308

BC, Backcross; DBH, Diameter at breast height; Max, Maximum; Min, Minimum; N, Number of trees sampled.

### Framework maps for QTL analysis

For QTL analysis, a framework of testcross (1:1) DArT markers were selected from the previously constructed, high-density genetic maps [37]. Using a minimum interval support of LOD 3.0, framework genetic linkage maps were produced for the *E. grandis* and *E. urophylla* BC parents and the F_1_ hybrid parent (Additional file 1: Table S1 and Additional file 2: Table S2). The average marker interval of the parental framework maps ranged from 6.1 cM (F_1_ hybrid in the *E. grandis* BC family) to 8.5 cM (*E. urophylla* BC parent). On average, 92 to 96% of loci in the four parental framework maps were within 10 cM of a neighbouring marker. The linkage group numbering and the orientation of the linkage groups were in accordance with Brondani *et al.*[38].

### QTL analysis of DBH and wood density

QTL analysis of DBH and wood density identified a total of five QTLs for DBH and 12 for wood density in the two backcross families (Tables 2 and 3). For DBH, two QTLs one on LG9 and one on LG10 were identified in the *E. grandis* and *E. urophylla* BC parental maps, respectively. The F_1_ hybrid contained one and two QTLs for DBH in the *E. urophylla* and *E. grandis* BC families, respectively (Table 2). There was a partial overlap of the DBH QTL on LG6 detected in the two F_1_ hybrid maps (Table 2, Additional file  3: Figure S1). The percentage of DBH variation explained by these QTLs individually ranged from 4.6 to 8.0% (Table 2). The positive effects of the DBH QTLs in the F_1_ hybrid maps were associated with the presence of the *E. grandis* allele, except for the QTL on LG6, which was associated with the *E. urophylla* allele.

**Table 2:** **Putative QTLs for DBH at age four years identified by CIM in the*E. grandis*and*E. urophylla*backcross mapping families**

**Parental map**	**Linkage group**	**cM position**	**LOD**	**Variance (R**^**2**^**) explained by QTL (%)**	**Additive effect (SD)**^**a**^
*E. grandis* BC parent	9	35.9	3.4	5.5	0.50
F_1_ hybrid parent (*E. grandis* BC family)	6	32.9	3.2*	5.0	−0.48
	10	89.0	5.9**	8.0	0.63
F_1_ hybrid parent (*E. urophylla* BC family)	6	51.0	4.3*	6.6	0.52
*E. urophylla* BC parent	10	3.0	4.6*	4.6	0.40

BC, Backcross; CIM, Composite interval mapping; DBH, Diameter at breast height; QTL, Quantitative trait locus; R^2^, Percentage of variance explained; SD, Standard deviation; * Significant at genome-wide α = 0.05; ** Significant at genome-wide α = 0.01.

^a^ Marker data in the F_1_ hybrid maps were recoded so that positive and negative additive effect values on all linkage groups are associated with the effect of replacement of the *E. grandis* allele with the *E. urophylla* allele in backcross progeny. Linkage phases were arbitrary from one linkage group to the next for the backcross parents and the directions of the effects are therefore not indicated for these parents.

**Table 3:** **Putative QTLs for wood density at four years identified by CIM in the*E. grandis*and*E. urophylla*backcross mapping families**

**Parental map**	**Linkage group**	**cM position**	**LOD**	**Variance (R**^**2**^**) explained by QTL (%)**	**Additive effect (SD)**^**a**^
F_1_ hybrid parent (*E. grandis* BC family)	1	76.3	2.5	3.1	−0.46
	4	12.5	3.9*	5.5	−0.56
	9	41.5	2.9	4.2	−0.52
F_1_ hybrid parent (*E. urophylla* BC family)	2	99.2	2.9	3.3	−0.36
	3	82.0	2.9	3.3	−0.36
	4	39.7	8.2**	8.2	0.62
	6	20.1	4.4**	5.6	−0.50
	8	75.7	2.9	3.3	−0.36
	9	65.5	11.1**	12.2	0.70
	10	16.5	4.0**	6.2	0.53
	10	58.8	8.2*	10.8	0.66
*E . urophylla* BC parent	8	32.0	2.6	5.0	0.46

BC, backcross; CIM, Composite interval mapping; DBH, Diameter at breast height; QTL, Quantitative trait locus; R^2^, percentage of variance explained; SD, Standard deviation; * Significant at genome-wide α = 0.05; ** Significant at genome-wide α = 0.01.

^a^ Marker data in the F_1_ hybrid maps were recoded so that positive and negative additive effect values on all linkage groups are associated with the effect of replacement of the *E. grandis* allele with the *E. urophylla* allele in backcross progeny. Linkage phases were arbitrary from one linkage group to the next for the backcross parents and the directions of the effects are therefore not indicated for these parents.

Between one and eight QTLs were identified for wood density (Table 3). Only one QTL for density was identified in the *E. urophylla* BC parent and no QTLs were identified in the *E. grandis* BC parent. The majority of the density QTLs were identified in the F_1_ hybrid maps, with three and eight QTLs detected in the *E. grandis* and *E. urophylla* BC families, respectively (Table 3, Additional file 3: Figure S1). The percentage of phenotypic variation explained by the density QTLs ranged from 3.1 to 12.2% (Table 3). The positive effects of all three QTLs identified for density in the F_1_ hybrid map of the *E. grandis* BC family were associated with the *E. urophylla* allele. For the F_1_ hybrid map of the *E. urophylla* BC family, positive effects of QTLs on LG4, 9 and 10 were associated with the *E. grandis* allele, while those on LG2, 3, 6 and 8 were associated with the *E. urophylla* allele. Analysis of epistatic interactions among DBH and wood density QTLs revealed two significant interactions, one between LG2 and LG8, one between LG8 and LG10 for wood density QTLs in the F_1_ hybrid map of the *E. urophylla* BC family (Additional file 4: Figure S2).

### Transcriptome profiling and expression QTL mapping of genes underlying a major QTL interval for wood density

**Figure 2:**
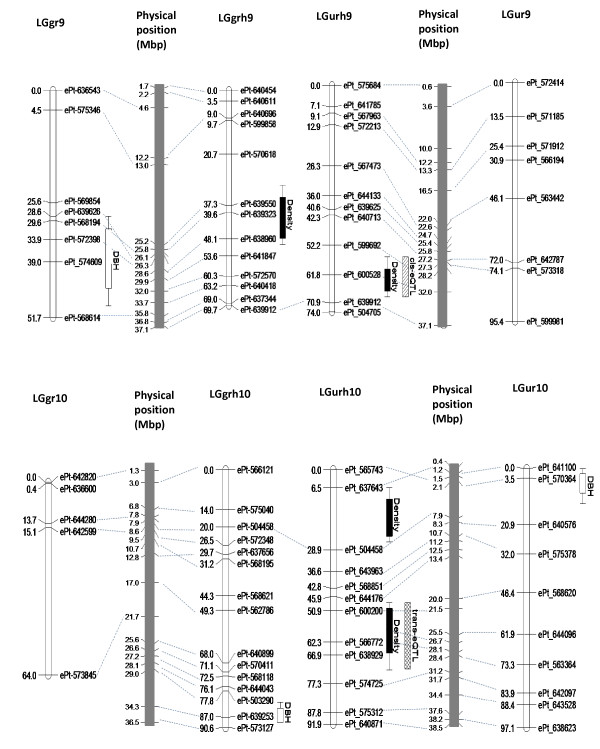
**Comparative QTL mapping of DBH and wood basic density at four years of age in the*E. grandis*,*E. urophylla*and F**_**1**_**hybrid parents of the F**_**2**_**backcross families.** Location of putative QTLs associated with DBH (white box) and wood density (black) on linkage group 9 and 10. A major QTL for wood density was found on chromosome 9 in the F_1_ hybrid in the *E. urophylla* BC family. Variation in transcript abundance of genes located in the QTL interval, which were also correlated with wood density (Table 4), were explained in part by the presence of shared trans-eQTLs, which co-localized with the wood density QTL on linkage group 10 (white boxes with cross hashing), in addition (for some genes) cis-eQTLs co-locating with the wood density QTL on LG 9 (white boxes with hashing). The backcross and F_1_ hybrid parental maps are connected by dotted lines through the physical position of the DArT marker fragments in the *E. grandis* genome sequence (V1.0 assembly, http://www.phytozome.net/). Map positions in centiMorgan (cM Kosambi) and megabase-pair (Mbp) are shown for the genetic and physical maps, respectively. The F_1_ hybrid maps constructed for the two backcross families are connected through shared testcross markers that segregated in both backcrosses. The position (solid bars, 95% CI; lines, 90% CI) of QTLs detected using composite interval mapping (CIM) are projected onto the genetic maps.

A QTL for wood density on LG9 (LOD support = 11.1, std additive effect = 0.70) of the F_1_ hybrid map for the *E. urophylla* BC family explained 12.2% of the phenotypic variation (Table 3). To identify the corresponding QTL interval on the genome sequence, the DNA sequences of flanking DArT markers (ePt_599692-ePt_639912) were used in a BLAST search against the *E. grandis* genome sequence and were found to be located at positions 28,269,541 and 37,191,970 on chromosome scaffold 9 (*E. grandis* genome assembly V1.0, http://www.phytozome.net). A total of 719 annotated genes were found in the QTL interval (Additional file 5), of which transcripts were detected by Illumina RNA-Seq analysis for 474 (65.9%) genes in the immature xylem of at least 25% of the sampled (*n =* 96) individuals of the *E. urophylla* BC family. The transcript abundance data for these 474 genes were used for expression QTL (eQTL) analysis. The population wide mean of the FPKM values for the 474 genes in the wood density QTL interval was 1,175,774.0 with standard deviation of 299,639.5. The FPKM values ranged between 0 and 34,859,400. Of the 474 genes (FPKM values) used for the eQTL analysis, approximately 15% were not normally distributed, but showed bimodal distribution suggestive of a single large effect eQTL (results not shown). A total of 371 eQTLs were identified for 294 (62%) of the xylem expressed genes and 63 (21.4%) of the genes had more than one eQTL. The transcript variation of the majority (70.8%) of these genes was associated with trans-acting polymorphisms (trans-eQTLs, Additional file 6: Figure S3), i.e. the eQTLs mapped outside of the trait QTL interval on LG9, or on a different linkage group. Only 86 genes in the interval had cis-acting eQTLs overlapping the gene positions. Genes with transcript abundance that is highly correlated with overall trait variation can be considered potential candidate genes [39], which provides a way to narrow down the number of candidates for further study. Of the 474 xylem expressed genes in the QTL interval, the transcript abundance of ten genes was correlated at *R*^*2*^ > 0.4 with wood density (Table 4). eQTL analysis for these ten genes revealed that four and seven of the genes shared trans-acting eQTLs on LG4 (10–33 cM) and LG10 (48–79 cM), respectively (Table 4). The positions of the trans-eQTLs detected on LG10 overlapped with that of a major wood density QTL detected in the same region of the linkage group (Figure 2, Table 3).

**Table 4:** **The top ten positional candidate genes located in the wood density QTL interval on linkage group 9 of the F**_**1**_**hybrid parent in the*E. urophylla*backcross family (52.2 cM, 28,269,541 bp to 70.9 cM, 37,191,970 bp,*E. grandis*genome assembly V1.0,**http://www.phytozome.net/**)**

**Gene identification**	**Physical position (bp)**	**At. identification**	**At. description**	**eQTL on linkage group**	**eQTL position (cM)**	**Correlation with wood density (R**^**2**^**)**
Eucgr.I01988.1	29,648,986	AT5G15410.1	Cyclic nucleotide-regulated ion channel family protein	4, 6, 10	12, 101, 51	0.53
Eucgr.I02335.1	33,854,742	AT2G17820.1	Histidine kinase 1	4, 9, 10	27, 52, 74	0.52
Eucgr.I02215.1	32,019,312	AT5G65980.1	Auxin efflux carrier family protein	2, 9	74, 71	0.51
Eucgr.I02312.1	33,682,442	AT1G68490.1	Unknown	9, 10	58, 74	0.48
Eucgr.I02402.1	34,626,812	AT3G50930.1	Cytochrome BC1 synthesis	4, 10	20, 79	0.48
Eucgr.I02479.1	35,623,844	AT2G46600.1	Calcium-binding EF-hand family protein	10	48	0.47
Eucgr.I02498.1	35,941,078	AT1G23780.1	F-box family protein	9	52	0.43
Eucgr.I02108.1	30,728,548	AT3G19660.1	Unknown	10	51	0.42
Eucgr.I02337.1	33,876,173	AT5G66180.1	S-adenosyl-L-methionine-dependent methyltransferases superfamily protein	9	67	0.41
Eucgr.I01935.1	29,248,252	AT5G39865.1	Glutaredoxin family protein	4, 10	33, 77	0.41

The positional candidate genes were identified using physical location, expression analysis and correlation of transcript abundance with wood density trait variation.

At, *Arabidopsis thaliana*; eQTL, Expression quantitative trait locus; Eucgr., *Eucalyptus grandis*; QTL, Quantitative trait locus.

**Figure 3:**
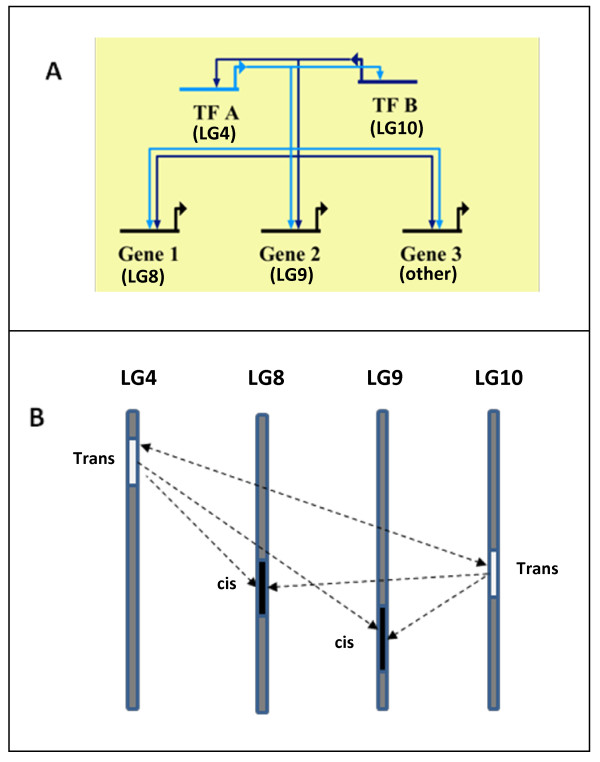
**Gene and map based models for the putative modes of action of eQTLs and trait QTLs.A**. Putative gene-based transcriptional network explaining the relationship of trans-acting eQTLs (transcription factors, TFs) and cis-eQTLs (in target genes) observed at the corresponding trait QTLs. In this model, cis-trans and trans-trans interactions may be possible at the gene and protein levels. **B**. Map-based model showing the putative interactions of QTLs for wood basic density. In this model, trans-acting eQTLs on LG4 and LG10 (white bars) underlie the wood basic density QTLs observed on these linkage groups, while genes with cis-acting eQTLs on LG8 and 9 (black bars) underlie QTLs detected on the same linkage groups and share trans-eQTLs on LG4 and LG10. The cis-trans interaction (at the gene level) between these two sets of QTLs putatively underlie the detectable epistatic interaction of the wood density QTLs on linkage groups 8 and 10 (Additional file 4 Figure S2).

To further investigate the nature of the epistatic interaction detected for wood density QTLs on LG8 and LG10, we performed eQTL analysis of the top ten most correlated genes (transcript abundance positively correlated with wood density variation) in the QTL interval on LG8. As was the case for LG9, several of the genes shared trans-eQTLs on LG4 and LG10 (Additional file 7: Table S3a) which overlapped fully or in part with wood density QTLs on LG4 and LG10. These results imply that the underlying gene(s) at the wood density QTLs on LG8 and LG9 (and possibly also at other wood density QTLs) may be controlled in part by trans-acting genes underlying the wood density QTLs on LG4 and LG10. These genes (and the corresponding QTLs) may therefore be segregating components of an underlying transcriptional network regulating wood density in these trees (Figure 3). eQTL analysis of genes located in the LG8 and LG9 QTL intervals that were most negatively correlated (at the level of transcript abundance, Additional file 5) with wood density trait variation (Additional file 7: Table S3b) did not reveal a strong pattern of shared trans-eQTLs associated with other wood density QTLs as was observed for the positively correlated genes in these intervals. Instead, several of the negatively correlated genes had cis-eQTLs co-located with to the wood density QTLs on LG8 and 9.

## Discussion

While most previous QTL mapping studies of diameter growth and wood density in *Eucalyptus*[12,16-18,40,41] were based on F_1_ hybrid pedigrees, the present study focused on trait dissection in an F_2_ interspecific backcross pedigree. QTL mapping in the shared F_1_ hybrid parent and the *E. grandis* and *E. urophylla* BC parents allowed assessment of the architecture of interspecific as well as intraspecific genetic variation affecting trait variation. In this approach, fixed genetic differences between the parental species are likely to be in heterozygous state in the F_1_ hybrid and segregate in either or both backcross families depending on the degree of dominance. In addition, genetic factors that are heterozygous in the backcross parents (i.e. intraspecific variation) also segregate in the backcross progeny. If fixed genetic differences between the pure-species parents were purely due to additive genetic effects, the majority of QTLs in the F_1_ hybrid would segregate in both backcross families. However, the majority of QTLs in the two F_1_ hybrid maps were detected in only one of the two backcross families. Only one QTL for DBH on LG6 in the F_1_ hybrid parent was shared in both backcross families (Additional file 3: Figure S1). Failure to detect QTLs segregating from the F_1_ hybrid in both backcross families may be due to dominance effects (Additional file 8: Figure S4) playing a significant role in the expression of QTLs in alternative genetic backgrounds [42-44], or may be the result of epistatic interactions [45], or due to differences in QTL effects for the same alleles segregating in different genetic backgrounds (i.e. in the presence of a different set of segregating QTLs). For example, we identified a significant epistatic interaction between the wood density QTLs on LG8 and LG10, and between the wood density QTLs on LG2 and LG8 in the F_1_ hybrid map (*E. urophylla* BC family). This may explain why we detected the wood density QTLs on LG2 and LG8 in the *E. urophylla* BC family only (Additional file 3: Figure S1).

Overall, more QTLs were identified in the F_1_ hybrid parent (14) than in the backcross parents (3) for DBH (three compared to one in the BC parents) and density (eleven compared to one in the respective backcross parent, Tables 2 and 3). The majority of the positive QTL effects for DBH in the F_1_ hybrid were associated with the *E. grandis* allele and most of the positive QTL effects for density were associated with the *E. urophylla* allele. This is congruent with the expected interspecific and intraspecific genetic variation segregating in the backcross families (Table 1, Figure 1). The number of QTLs detected for DBH in *Eucalyptus* has generally been lower than that detected for wood density (Additional file 9: Table S4). The lower number of QTLs identified for DBH in this study (Tables 2 and 3) is consistent with published QTL reports, reflecting the lower heritability associated with growth traits compared to wood density in *Eucalyptus* and the limited statistical power to detect larger numbers of small effect QTLs. The well-described Beavis effect [19] certainly also applies in our study which means that some QTL effects listed in Tables 2 and 3 may be inflated and we fully expect that more QTLs of lower effect would be detected if our mapping populations were to be expanded.

DBH and density QTLs were detected in different regions of the genome (Additional file 3: Figure S1) suggesting that the two traits are affected by independent polymorphic loci in this pedigree. This is further supported by the low phenotypic correlation observed between DBH and wood density in the two backcross families (0.06 for *E. grandis* BC and −0.02 for *E. urophylla* BC). Freeman *et al.*[16] also identified independent QTLs for DBH and wood density in an F_2_ outbred pedigree of *E. globulus*. However, other QTL studies in *Eucalyptus* have identified co-located QTLs affecting DBH and wood density in addition to independent QTLs [12,40]. This could be explained by the occurrence of different polymorphisms affecting the two traits in each mapping pedigree resulting in different levels of correlation reported for DBH and density in previous studies [46-50]. The identification of independent QTLs for DBH and wood density in this study suggests that MAB could be used to improve growth and wood density simultaneously in this hybrid pedigree by selecting for combinations of QTL alleles with positive effects on DBH as well as wood density.

Comparative genetic mapping facilitates the identification of QTLs across different environments, ages and in different genetic backgrounds. Previous comparative genetic mapping studies in *Eucalyptus* suggested high levels of synteny and co-linearity among the genomes of eucalypt species [37,38,51,52] enabling the comparative analysis of QTLs in different species [14,16,17,40,41,51]. QTLs for DBH and wood density were detected on homologous linkage groups in the parental maps in this study (Additional file 3: Figure S1) and *E. globulus* and *E. nitens* linkage maps in previous studies (Additional file 10: Table S5 and Additional file 11: Table S6). QTLs identified for wood density on LG1, LG6 and LG10 of the F_1_ hybrid map may correspond to wood density QTLs previously identified on the same linkage groups in *E. globulus*[16], while QTLs identified for wood density on LG6 and LG8 of the F_1_ hybrid map may correspond to wood density QTLs identified in *E. nitens*[17]. A QTL identified for wood density on LG9 of the F_1_ hybrid map (*E. urophylla* BC) may correspond to a wood density QTL previously identified in *E. nitens*[17]. Similarly, QTLs identified for DBH on LG6 and LG10 (Table 2) of the same parental map may represent the same genomic regions as DBH QTLs reported in *E. nitens*[40] and *E. globulus*[16], respectively. Common regions affecting trait variation across species should be the priority targets for the identification of candidate genes, the development of gene-based markers, association genetic studies and eventually MAB. We expect the resolution of comparative QTL analysis to drastically improve with the use of large numbers of trans-specific and trans-pedigree markers such as microsatellite, DArT and SNP markers linked to the *E. grandis* reference genome sequence.

An advantage of MAB in trees is the early selection of seedlings, reducing the time and cost normally involved in growing trees to maturity in the field before being able to identify elite trees [10]. Experiments in crop plants have indicated that major effect QTLs and candidate genes associated with these QTLs are more reliable for MAB [53]. Most of the QTLs detected in previous studies in *Eucalyptus* have likely been from the top end of the distribution of segregating QTL effects, some of which could be considered major effect QTLs [12,14-17,40]. However, there is a bottleneck between mapped QTLs and gene discovery mainly due to the low resolution of QTL mapping in populations of only several hundred individuals. To extend the information of QTL mapping, genetical genomic approaches [26] have been used to identify positional candidate genes and regulatory networks underlying phenotypic variation in several plant species [13,22-24,27]. In this study, the majority (70.8%, Additional file 6: Figure S3) of eQTLs identified for 294 xylem expressed genes underlying a major wood density QTL on LG9 (F_1_ hybrid map, Figure 2, Table 3), did not co-locate with the physical positions of the genes (i.e. were trans-acting eQTLs). The trans-eQTLs detected for these genes most likely correspond to diverse regulatory factors controlling the expression of the genes located in the QTL interval on LG9 one (or more) of which could harbor the trait altering polymorphism underlying the wood density QTL. eQTLs co-localizing with the physical genome position of the gene (cis-eQTL; 29.2%) were identified for only 86 genes in the interval, which is in agreement with the lower proportion of cis-eQTL previously reported for *Eucalyptus* (22%) in an interspecific backcross population of *E. grandis* and *E. globulus*[54] and more recently for *Populus* (23%) using whole-genome microarray analysis in an interspecific hybrid pedigree [33].

Schadt *et al.*[39] reported that genes whose transcript levels are correlated with trait variation could be considered potential candidate genes for the trait. In the present study the transcript levels of ten genes located in the wood density QTL interval on LG9 were found to be positively correlated (*R*^2^ > 0.4) with wood density variation (Table 4). Some of these genes encoding a nucleotide-regulated ion channel family protein (DND1), histidine kinase (HK), S-adenosyl-L-methionine-dependent methyltransferase (SAM), an auxin efflux carrier family protein and calcium-binding EF-hand family protein, have previously been reported to be involved in plant growth and development and cell wall biogenesis. The transcript abundance of three of these genes (*DND1HK1* and a gene encoding a calcium binding EF-hand family protein) was affected by trans-acting eQTLs on LG10 (Table 4). Importantly, the same genomic region on LG10 (51 cM to 74 cM) co-localized with a major wood density QTL on the same linkage group suggesting that this genomic region may contain trans-acting factors affecting wood density as well as the transcript abundance of the candidate genes underlying the wood density QTL on LG9 (Figure 2). DND1 has been shown to be involved in plant defense responses in *Arabidopsis*[55]. HK was reported to act as a cytokinin receptor [56] involved in diverse plant growth and developmental processes [57,58]. SAM is a key enzyme for the phenylpropanoid pathway, involved in the synthesis of lignin [59]. Auxin, essential for plant growth and development (e.g. vascular tissue differentiation, apical development, cell elongation and tropical growth) is transported from cell to cell by auxin efflux carrier proteins [60-62]. Besseau *et al.*[63] showed that a reduction in the level of hydroxycinnamoyl-CoA shikimate/quinate hydroxycinnamoyl transferase (HCT), a gene involved in lignin biosynthesis, was correlated with the inhibition of auxin transport in *Arabidopsis*, suggesting that auxin efflux carrier family proteins might be important for cell wall deposition and lignification. Similarly, plant cells contain large amounts of calcium in their cell walls and previous studies highlighted Ca^2+^ playing a role in secondary cell wall biosynthesis [64,65].

The observation that the top most positively correlated genes (at the level of transcript abundance) in the wood density QTL interval on LG9 (Table 4) prominently shared trans-eQTLs on LG4 and LG10 (F_1_ hybrid map, *E. urophylla* BC family) suggested the presence of trans-acting factors that also underlie wood density QTLs at the same loci. This, together with the detection of a significant epistatic interaction between wood density QTLs on LG8 and LG10, led us to investigate the transcript abundance of genes in the QTL interval on LG8, with the hypothesis that a similar cis-trans relationship would exist between LG8 and LG10 as was observed for LG9 and LG10. We indeed found that the top most positively correlated genes in the wood density QTL interval on LG8 also shared trans-eQTLs on LG4 and LG10 (and LG6, Additional file 3: Table S3a). The top most negatively correlated genes in the wood density QTL intervals on LG 8 and 9 (Additional file 7: Table S3b) did not exhibit such a strong pattern of shared trans-acting eQTLs, but it is formally possible that any of the positively or negatively correlated genes in these two QTL intervals affect trait abundance via a cis-acting and/or trans-acting eQTLs. Together, these findings suggest that at least some of the wood density eQTLs detected in this study may represent segregating components of a transcriptional network (Figure 3). Furthermore, our results suggest that transacting genes (e.g. transcription factors) located in the QTL intervals on LG4 and LG10, together with putative target genes located in the QTL intervals on LG8, LG9 and other identified wood density QTLs should be prioritized for further investigation. Trans-acting factors for which the parental species are differentiated would be heterozygous in the F_1_ hybrid and could have large effect on gene expression and trait variation in backcross progeny. Cases where transcription factors as well as their target genes segregate may give rise to detectable epistatic interactions as was putatively observed for the wood density QTLs on LG10 (trans-acting) and LG8 (cis-acting).

## Conclusion

We have detected QTLs for DBH and wood density in an interspecific backcross pedigree of *E. grandis* x *E urophylla*, with each QTL explaining between 3.1 and 12.2% of the phenotypic variation. Furthermore, our study is the first to use Illumina RNA-Seq expression profiling in a segregating tree population to quantify transcript abundance and map eQTLs for candidate genes potentially affecting wood property variation. This approach allowed us to detect cis- and trans-eQTLs for candidate genes co-locating with four wood density QTLs generating a hypothesis for the underlying mode of action of the trait QTLs and suggesting the presence of a transcriptional network of which some components may segregate in this backcross pedigree (Figure 3). Candidate gene-based markers developed from these QTL and eQTL regions will promote MAB of hybrids of *E. grandis* and *E. urophylla* and allow more detailed molecular genetic dissection of quantitative trait variation in these trees.

## Methods

### Plant materials

Two previously described [37] interspecific F_2_ backcross families sharing an *E. grandis* x *E. urophylla* F_1_ hybrid parent (GUSAP1, Sappi, South Africa) were analysed to detect QTLs for DBH and wood basic density in the F_1_ hybrid and two pure-species, backcross parents. The F_1_ hybrid was originally derived from an *E. grandis* seed parent (GSAP1) pollinated with an *E. urophylla* pollen mix (parent unknown). A single F_1_ individual (GUSAP1) was selected for backcrossing to unrelated individuals of *E. grandis* (GSAP2) and *E. urophylla* (USAP1) to avoid possible inbreeding depression in the backcross progeny. A total of 308 seedlings of the *E. urophylla* BC family were planted in 2005 and 286 seedlings of the *E. grandis* BC family were planted in 2006 near KwaMbonambi, KwaZulu-Natal (Sappi, South Africa). The site chosen for the trial is located on flat coastal land with deep sandy soils and little spatial variation. The trees were planted in 2.7 x 2.2 m spacing and standard silvicultural operations were applied to ensure that the site was weed free at planting and remained weed free until canopy closure to ensure that the trees were not subjected to any competing vegetation.

### Phenotypic measurements

Diameter (cm) at breast height (DBH) of the main stem was assessed in the *E. urophylla* and *E. grandis* BC families at age four in 2009 and 2010, respectively. For the assessment of wood property traits, a wood disk taken at the height of 1.35 m on the main stem was used to determine wood basic density using the water displacement method [66].

### DNA isolation and DArT genotyping

High-throughput DNA extraction and DArT genotyping were used to obtain molecular marker genotypes for the three parental trees and all of the backcross progeny. The *Eucalyptus* DArT array was the same as that developed by Sansaloni et al. [8] and subsequently used for genotyping in other studies [37,52]. The array comprises 7680 informative, polymorphic DArT markers selected by generating genomic representations from diverse *Eucalyptus* species and performing segregation analyses of more than 20,000 DArT polymorphisms in various *Eucalyptus* mapping populations [8].

### Genetic mapping and QTL analysis

High-density, single-tree, genetic linkage maps were previously constructed for the *E. grandis* BC parent, the *E. urophylla* BC parent and the shared F_1_ hybrid parent using the two-way pseudo-testcross mapping strategy [37,67]. For QTL analysis, particularly for Composite Interval Mapping (CIM), it is desirable to have uniform spacing of marker loci [68] and there is not much to gain from having markers closer than 5–10 cM [69]. Therefore a subset of testcross DArT markers (1:1) with approximately 5–10 cM spacing were selected for the construction of framework linkage maps for the individual parental trees (Additional file 1: Table S1 and Additional file 2: Table S2). The map distances for the framework maps were recalculated using the Kosambi mapping function in Joinmap® 4 [70]. The parameter settings used were Rec = 0.40, LOD = 3.0 and Jump = 5.

Marker data in the F_1_ hybrid parent map were recoded (1-*E. grandis*, 0-*E. urophylla*), based on the genotype of the *E. grandis* parent of the F_1_ hybrid [37] so that the QTL effect at each locus could be associated with the *E. grandis* or *E. urophylla* allele inherited from the F_1_ hybrid. The directions of QTL effects for the *E. grandis* and *E. urophylla* BC parents are arbitrary from one linkage group to another with respect to the grandparent alleles.

QTL analysis was conducted using CIM according to Zeng [71] using WinQTL Cartographer 2.5 [72]. CIM was performed using Model 6 after scanning the genetic map and estimating the likelihood of a QTL and its corresponding effect at every 2 cM map interval, while using significant marker cofactors to adjust for the phenotypic effects associated with each target interval in the genetic map. Forward and backward stepwise regression, with a threshold of *p* <0.1, was used to select marker cofactors for background control with a window size of 10 cM on either side of the QTL test site. QTL peaks separated by a minimum distance of 20 cM or more on the same linkage group were considered evidence for two different QTLs [73]. For peaks within 20 cM, the highest peak was chosen to approximate the position of the QTL. QTLs were declared significant by comparing LOD scores to an empirical genome-wide significance threshold calculated from 1,000 permutations for genome-wide *α* = 0.05 and *α* = 0.01 to control for type-I error. All QTLs with LOD support greater than 2.5 were also recorded. QTL position, LOD support, coefficients of determination (*R*^2^) and additive effects were estimated for each QTL. We used QTLNetwork (V2.0) [74] with a mixed-model composite interval mapping approach to test for epistatic interactions among the observed QTLs for DBH and wood density. The identified QTLs were projected onto linkage maps using MapChart 2.1 software [75].

### Transcriptome profiling and eQTL analysis

The outer, differentiating xylem tissue from 96 individuals of the *E. urophylla* BC family was collected immediately after removing the bark from the zone of 1.5 to 2.0 m on the main stem. Tissue was immediately frozen in liquid nitrogen to avoid RNA degradation. The samples were stored at −80 °C until RNA extraction. Total RNA was isolated as described previously [76] and used for Illumina RNA-Seq analysis by BGI Americas (http://bgiamericas.com). A minimum of 20 million mapable paired-end reads (PE50) per individual was obtained. After quality filtering (per base Phred score > 20), on average 96% of the reads mapped (75% mapped as proper pairs, the rest as single-reads) to predicted gene models in the draft *E. grandis* genome sequence (DOE-JGI V1.0, http://www.phytozome.net) using TopHat version 1.30 [77] and gene expression values (fragments per kilobase of coding sequence per million mapped fragments, FPKM) were calculated for each predicted gene model using Cufflinks version 1.0.3 (bias correction and quartile normalization was enabled for the FPKM calculation) [78]. To identify putative positional candidate genes, eQTL analysis was performed for the genes underlying a major QTL interval for wood basic density on LG9 using CIM as described above for trait QTLs.

## Authors’ contributions

MMD and ARKK constructed the framework genetic maps. ARKK carried out the laboratory work, QTL and eQTL mapping and drafting of the manuscript. MMD contributed to the QTL analysis and edited the manuscript. CAH performed the transcriptome data analysis. NJ and AK contributed to the design of the study and the development of the cross and mapping pedigree, and edited the manuscript. AAM conceived and supervised the study as well as the drafting of the manuscript. All authors have read and approved the final version of the manuscript.

## Supplementary Material

Additional file 1 **Table S1.** Summary of the framework linkage maps for the *E* grandis BC family. Click here for file

Additional file 2 **Table S2. **Summary of the framework linkage maps for the *E urophylla* BC family. Click here for file

Additional file 3 **Figure S1.** Comparative QTL mapping of the *E. grandis*, *E. urophylla* and their F_1_ hybrid. Click here for file

Additional file 4 **Figure S2. **Epistatic interaction identified between LG8 and 10. Click here for file

Additional file 5 Gene expression values and genotypic data used for eQTL analysis. Click here for file

Additional file 6 **Figure S3. **eQTLs for the genes underlying a major wood density QTL. Click here for file

Additional file 7 **Table S3. **eQTLs identified for genes on LG8 and LG9. Click here for file

Additional file 8 **Figure S4. **Additive and dominance effects segregating in F2 progeny. Click here for file

Additional file 9 **Table S4. **QTLs studies reported for DBH and wood density in *Eucalyptus.* Click here for file

Additional file 10 **Table S5. **Comparative QTL analysis for DBH among different *Eucalyptus* species. Click here for file

Additional file 11 **Table S6. **Comparative QTLs for wood density among different *Eucalyptus* species. Click here for file
