# Supplementary material for: Genetic dissection of growth, wood basic density and gene expression in interspecific backcrosses of Eucalyptus grandis and E. urophylla
Source: BMC Genet. 2012 Jul 20;13:60. doi: 10.1186/1471-2156-13-60 (PMC3416674; doi:10.1186/1471-2156-13-60)

**Electronic supplementary material: Supplementary Figure 1**

**Title:** Genetic dissection of growth, wood basic density and gene expression in interspecific backcrosses of *Eucalyptus grandis* and *E. urophylla*

**Journal name:** BMC Genetics

**Authors:** Anand R.K. Kullan, Maria M van Dyk, Charles A. Hefer, Nicoletta Jones, Arnulf Kanzler, Alexander A. Myburg*

**Affiliation and e-mail address of corresponding author:**

Department of Genetics, Forestry and Agricultural Biotechnology Institute (FABI), University of Pretoria, Pretoria, 0002, South Africa

zander.myburg@fabi.up.ac.za

**Supplementary Figure 1**. Framework linkage maps of the *E. grandis* (gr), *E. urophylla* (ur) and F_1_ hybrid parent of the *E. grandis* (grh) and *E. urophylla* (urh) backcross families showing the location of putative QTLs associated with DBH (white vertical bars) and wood basic density (black vertical bars). The backcross and F_1_ hybrid parental maps are connected by dotted lines using the physical position of the DArT marker fragments in the draft (V1.0) *E. grandis* genome sequence (http://www.phytozome.net/). Map positions in centiMorgan (cM Kosambi) and megabase-pair (Mbp) are shown for the genetic and physical maps, respectively. The F_1_ hybrid maps constructed for the two backcross families are connected through shared testcross markers that segregated in both backcrosses. Positions (solid bars, 95% CI; lines, 90% CI) of QTLs detected using composite interval mapping (CIM) are projected onto the genetic maps.

**LGgr1**

**LGgrh1**

**LGurh1**

**LGur1**

**Physical position**

**Physical position**


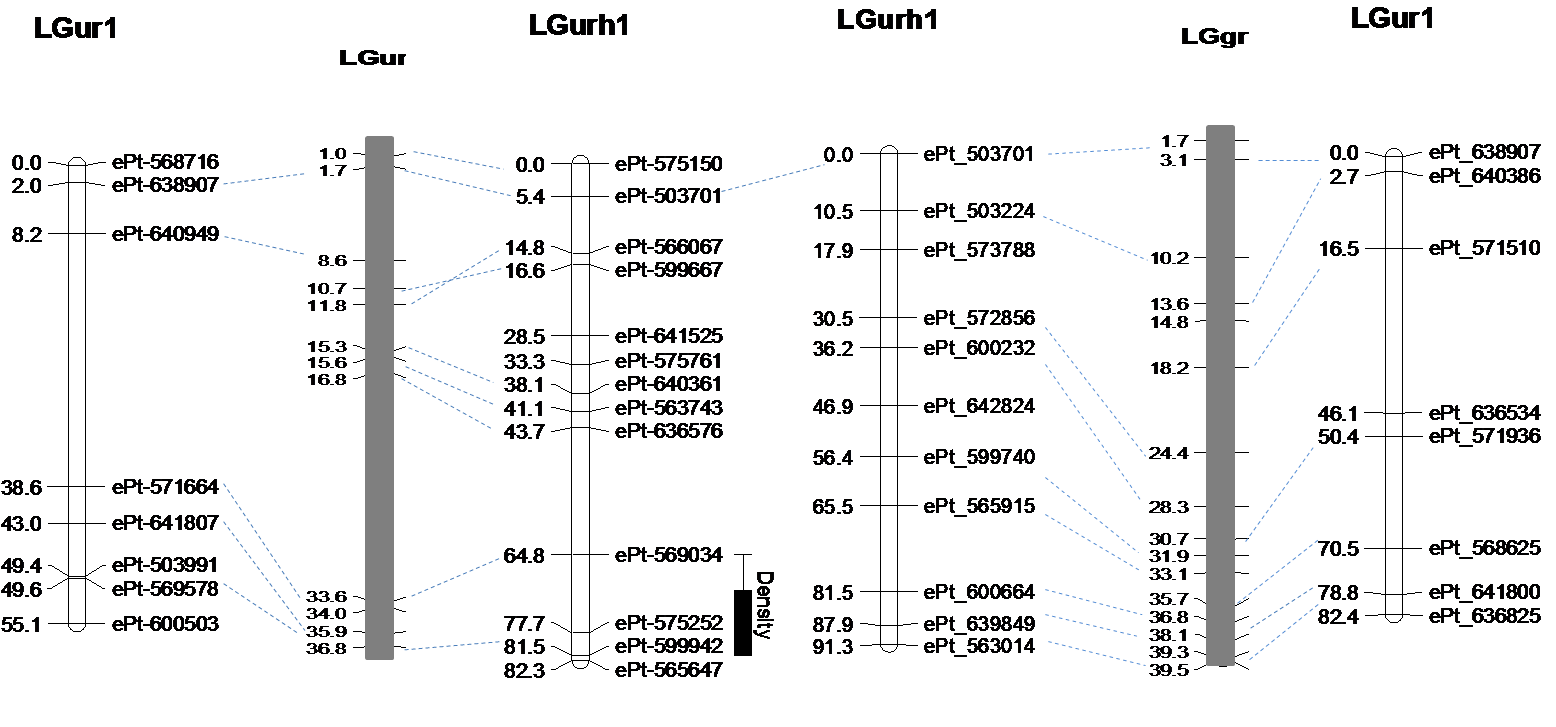


**LGgr2111**

**LGgrh2**

**LGurh2**

**LGur2**

**Physical position**

**Physical position**


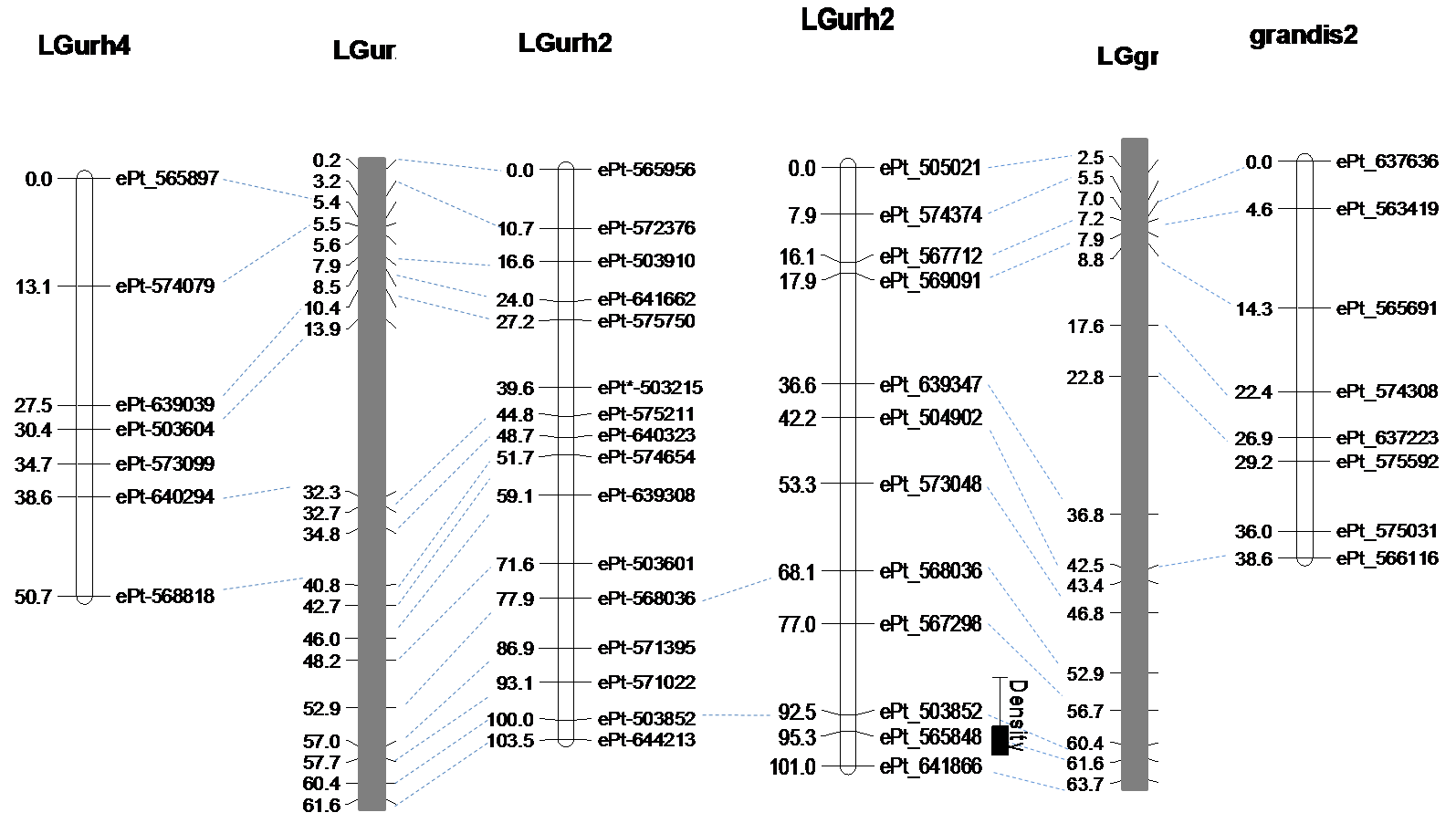


**LGgr3**

**LGgrh3**

**LGurh3**

**LGur3**

**Physical position**

**Physical position**


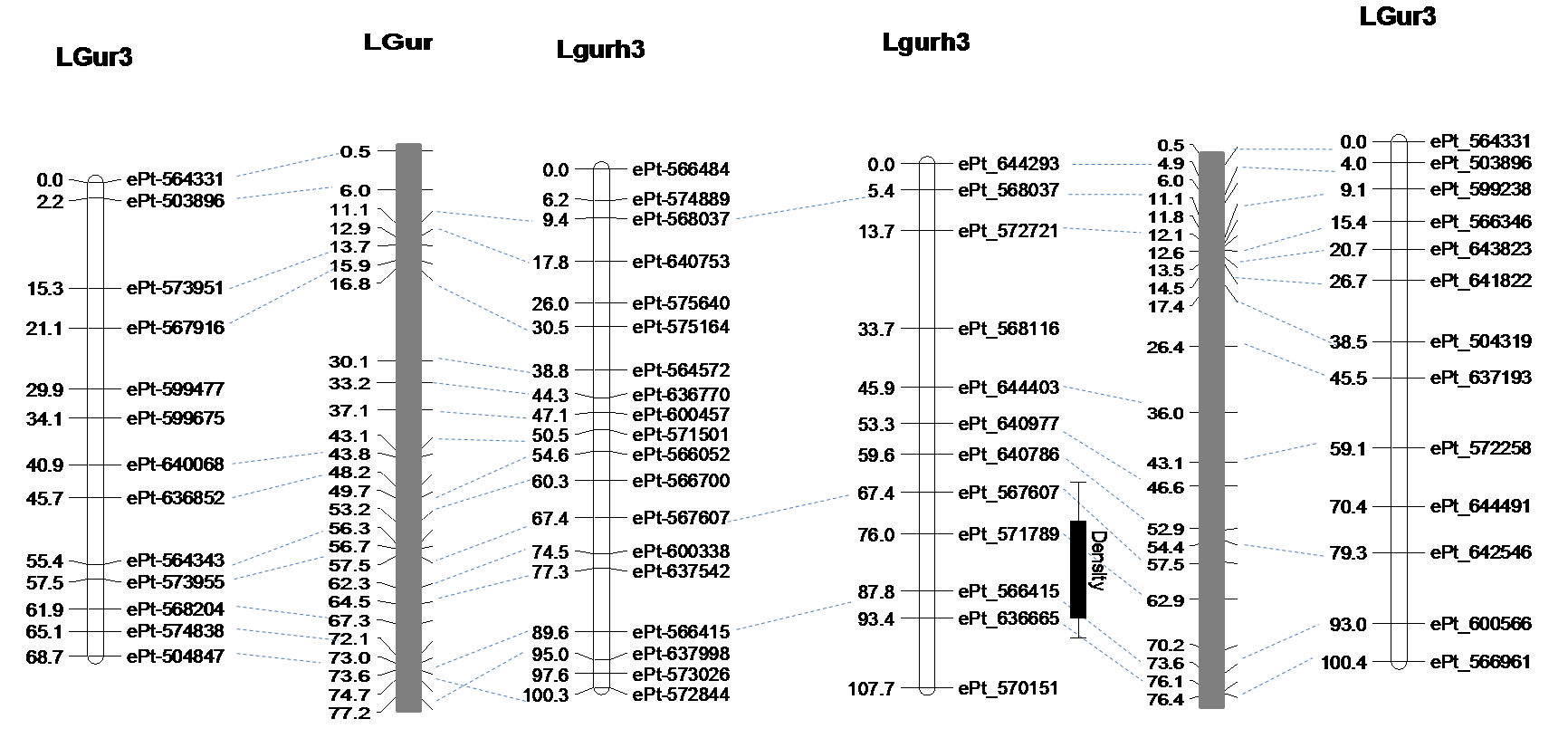


**LGgr4**

**LGgrh4**

**LGurh4**

**LGur4**

**Physical position**

**Physical position**


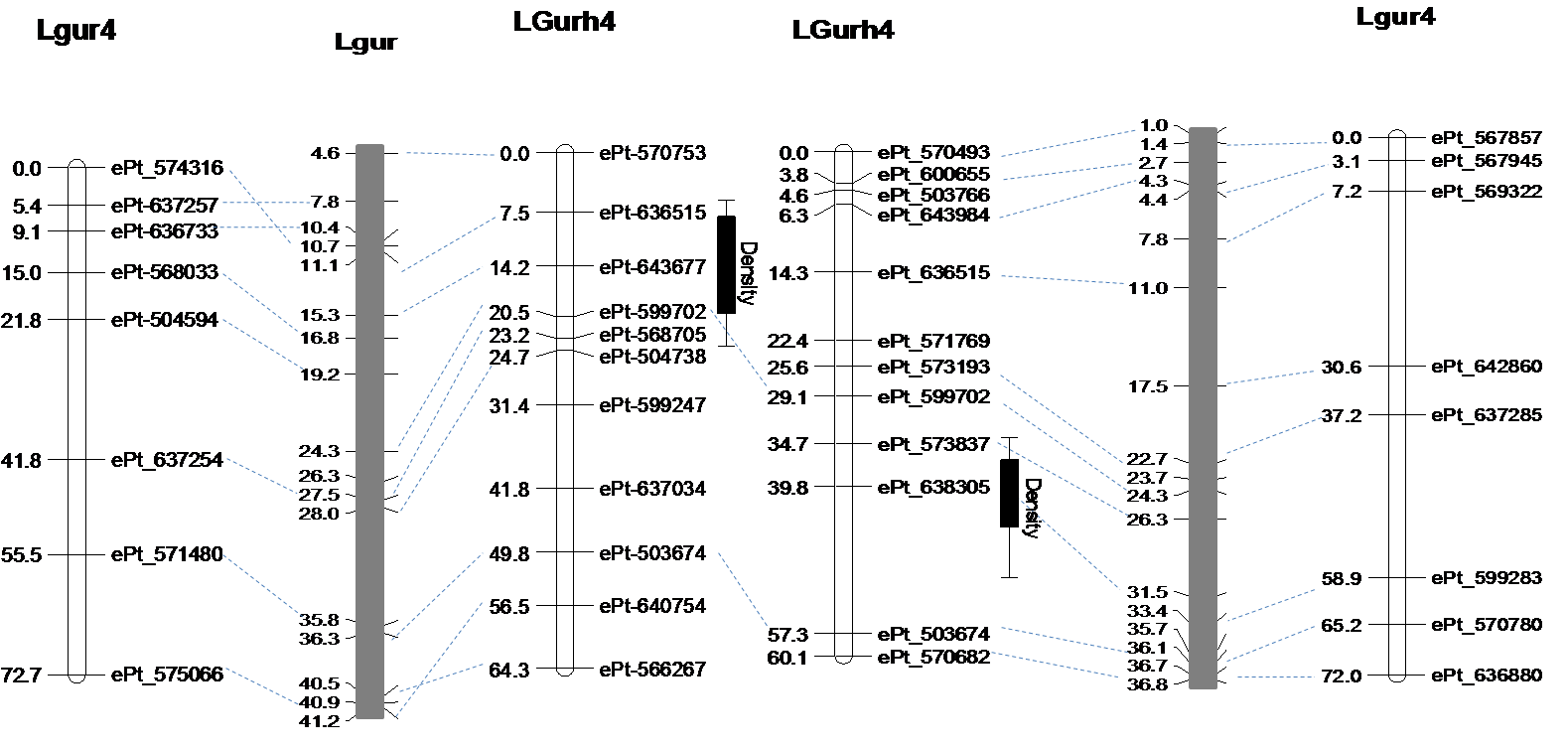


**LGgr5**

**LGgrh5**

**LGurh5**

**LGur5**

**Physical position**

**Physical position**


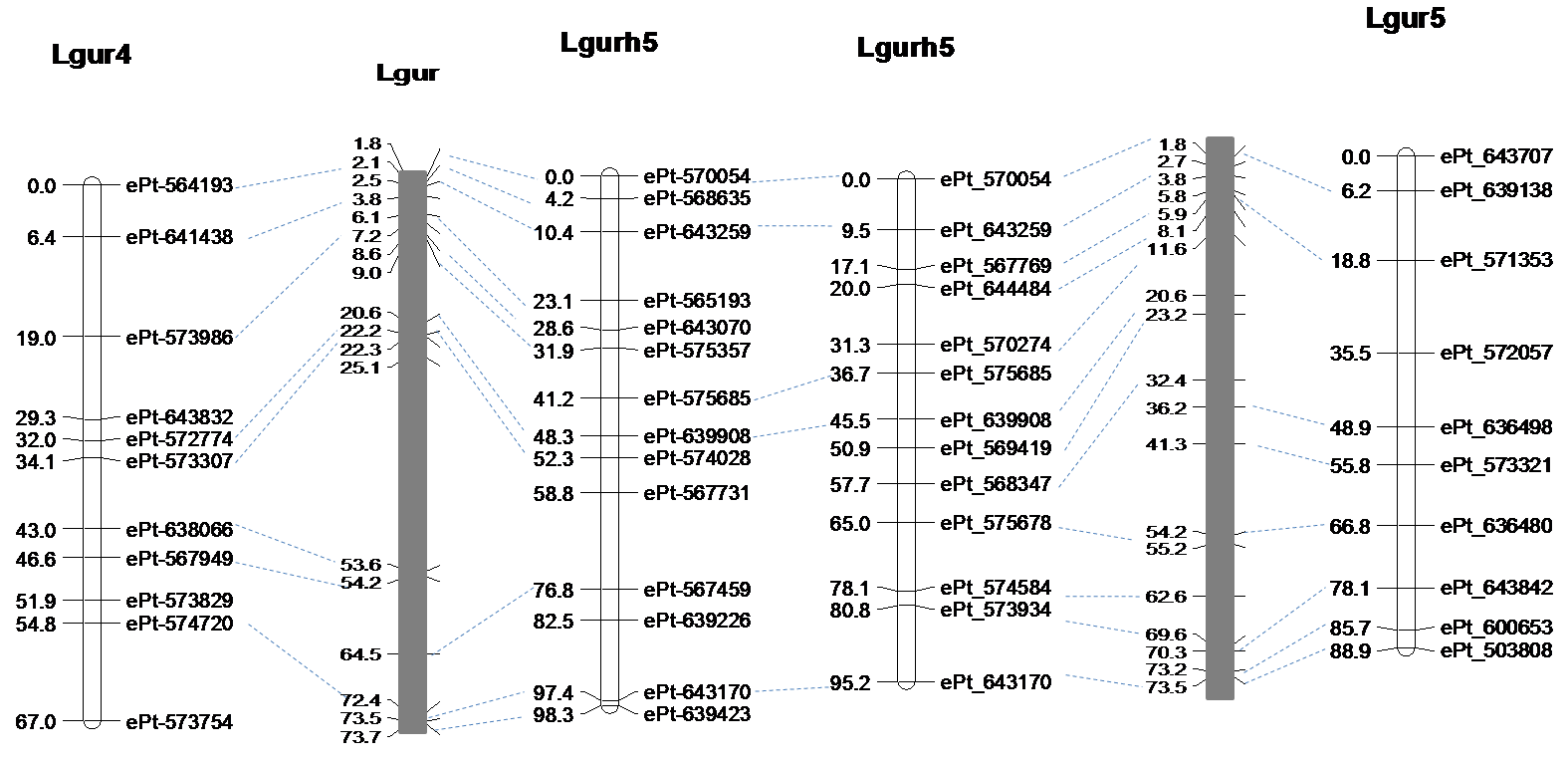


**LGgr6**

**LGgrh6**

**LGurh6**

**LGur6**

**Physical position**

**Physical position**


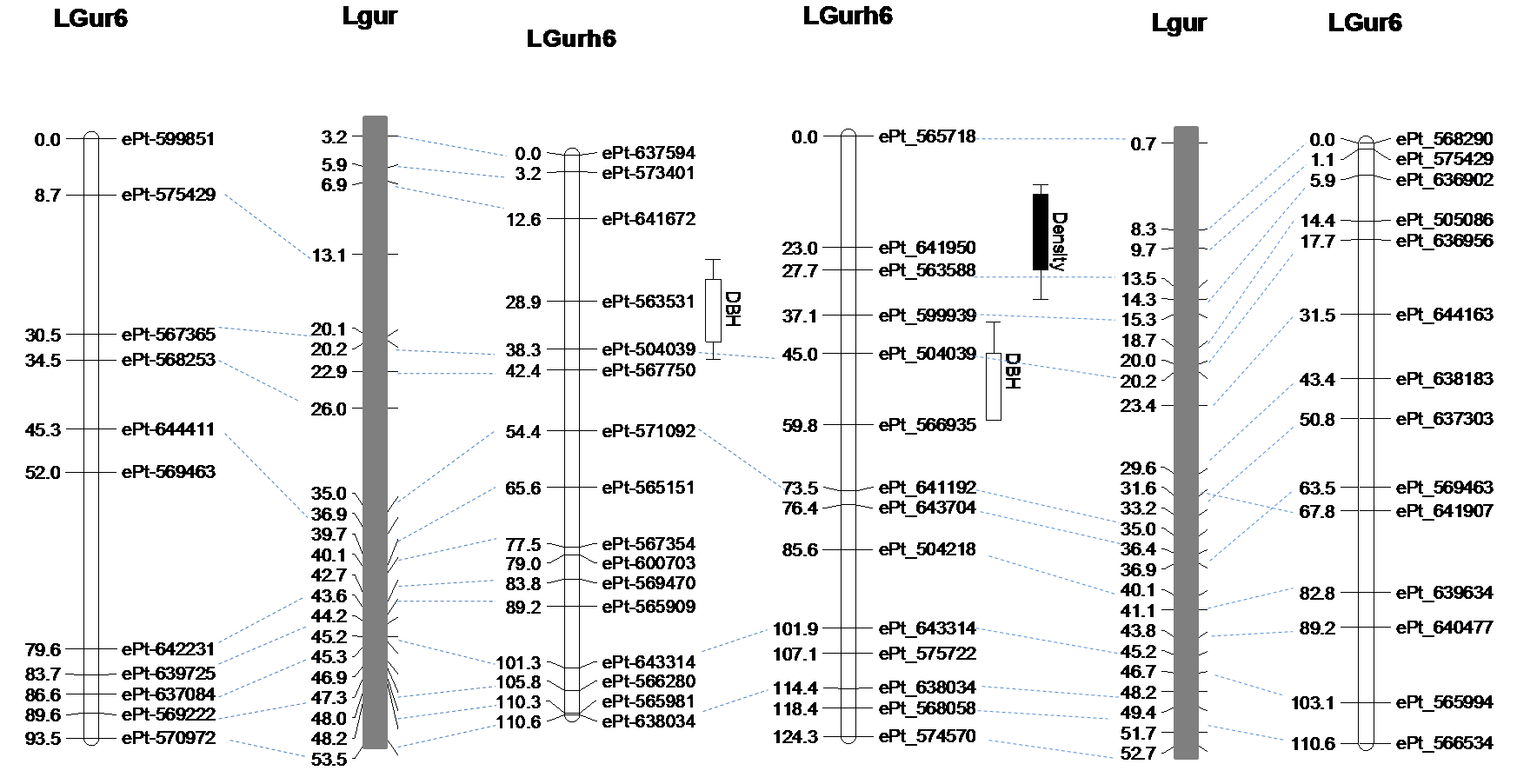


**LGgr7**

**LGgrh7**

**LGurh7**

**LGur7**

**Physical position**

**Physical position**


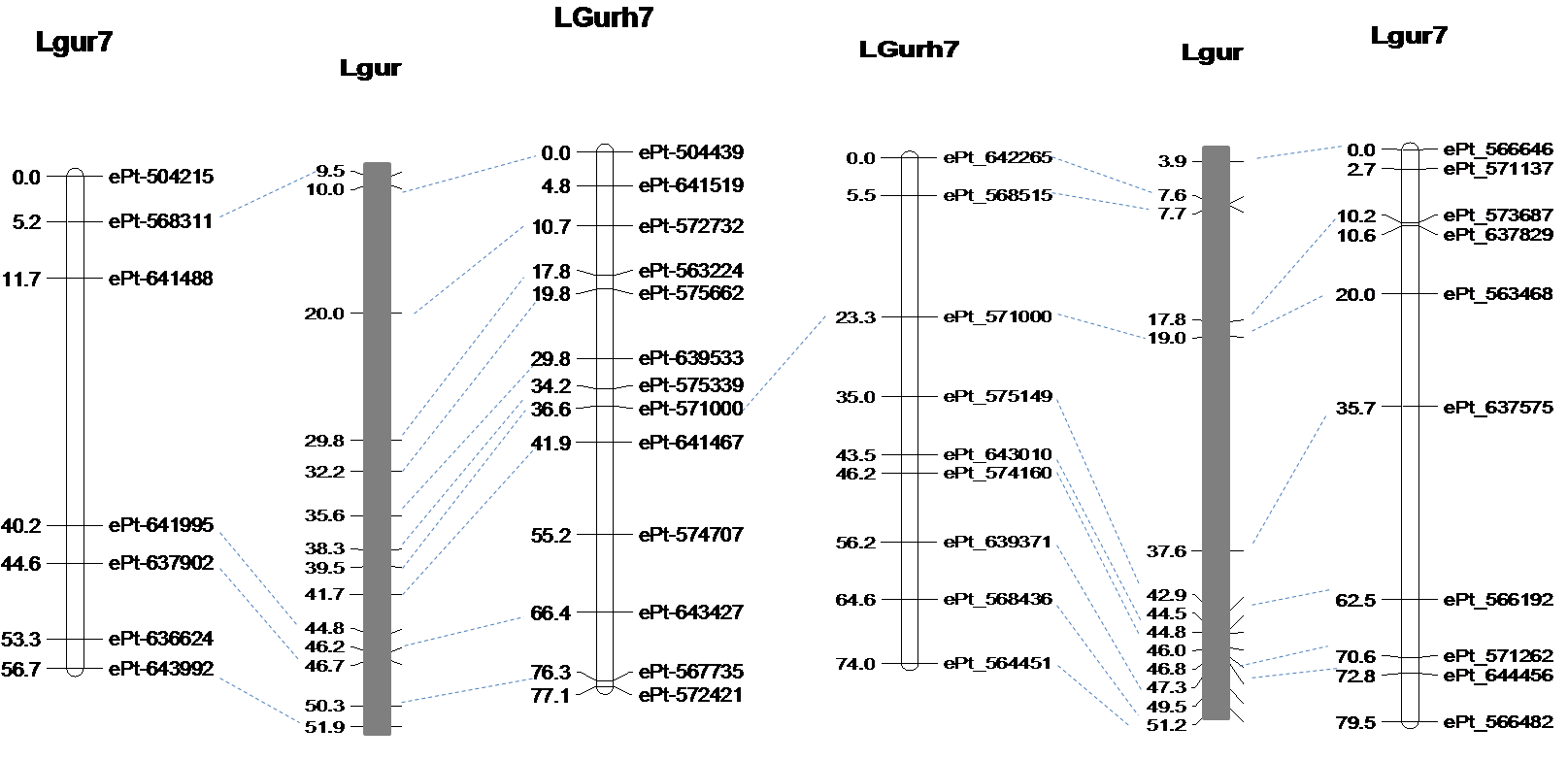


**LGgr8**

**LGgrh8**

**LGurh8**

**LGur8**

**Physical position**

**Physical position**


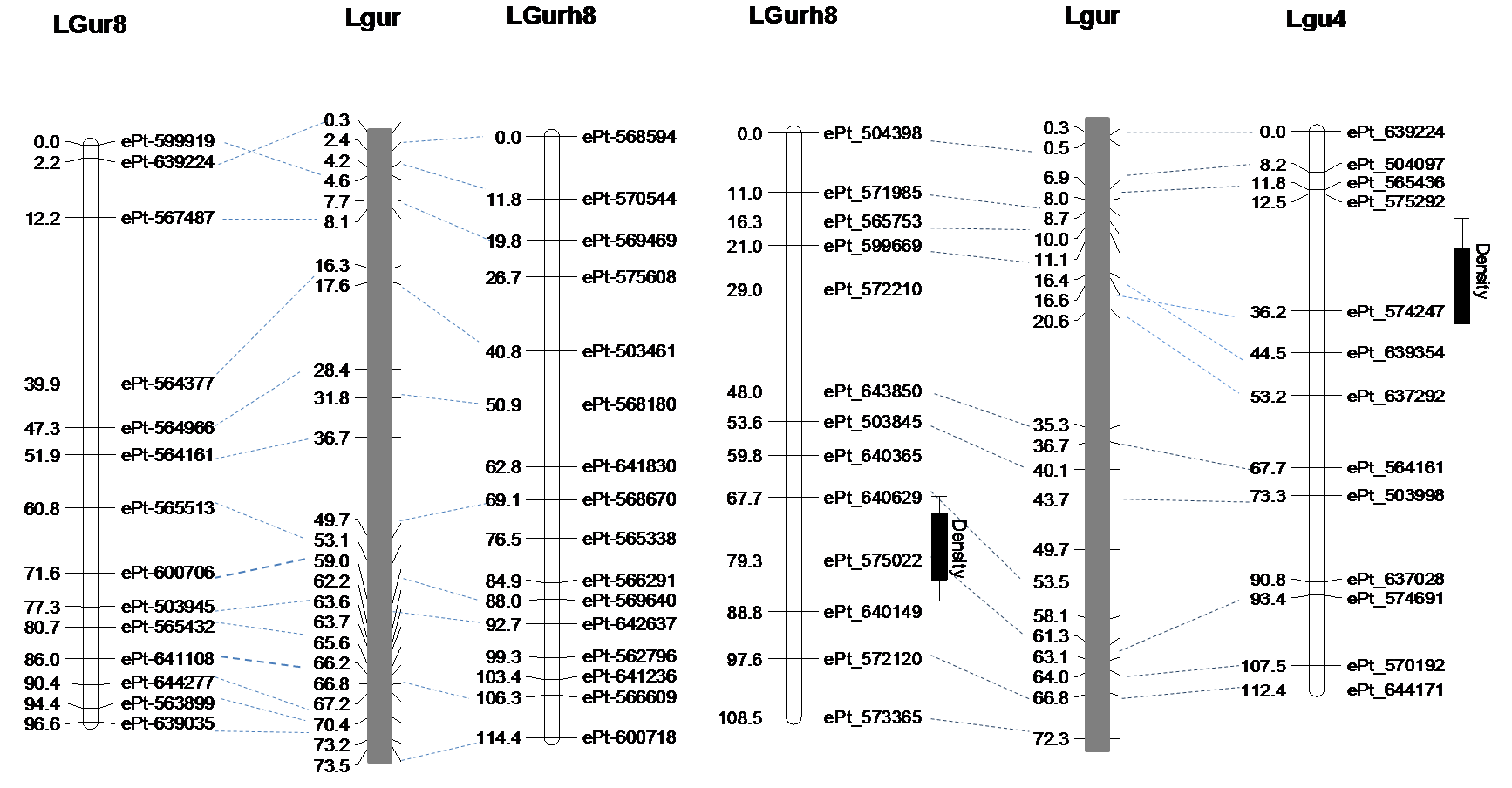


**LGgr9**

**LGgrh9**

**LGurh9**

**LGur9**

**Physical position**

**Physical position**


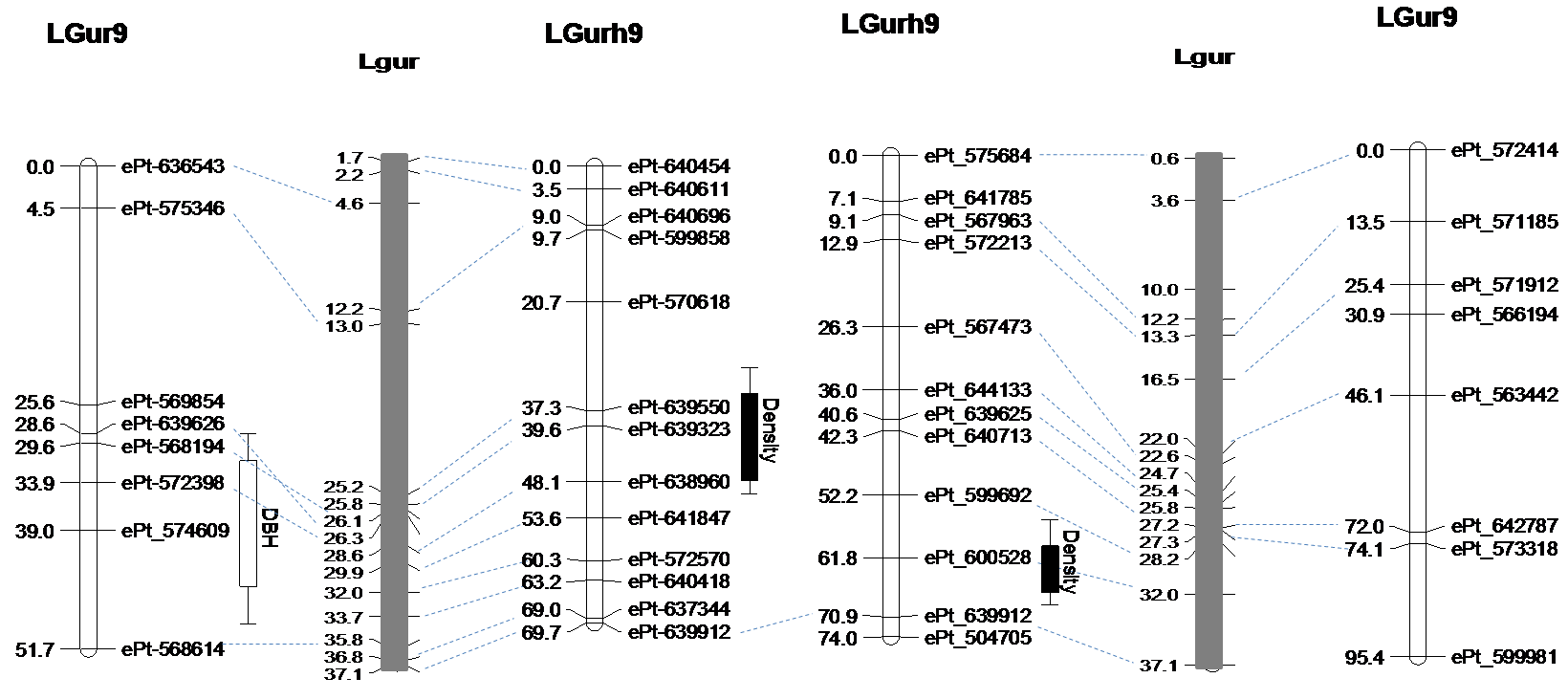


**LGgr10**

**LGgrh10**

**LGurh10**

**LGur10**

**Physical position**

**Physical position**


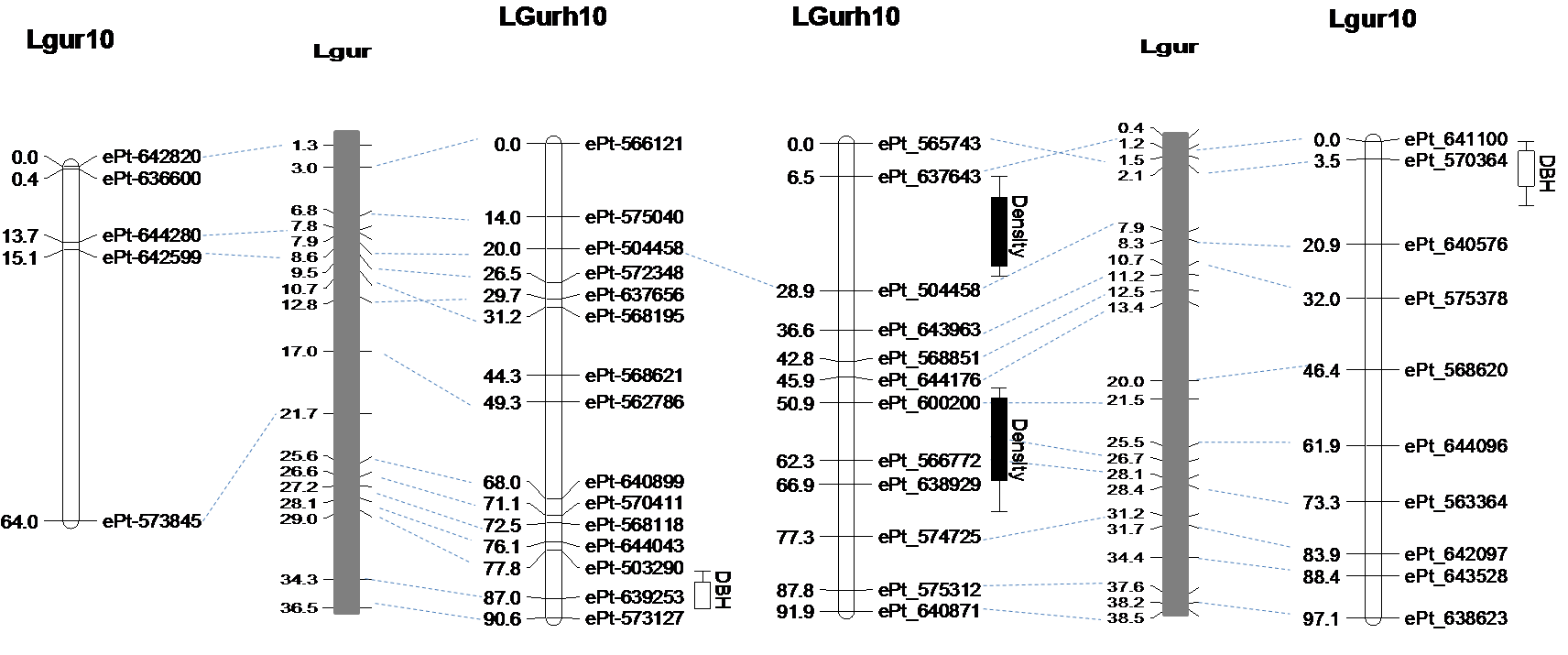


**LGgr11**

**LGgrh11**

**LGurh11**

**LGur11**

**Physical position**

**Physical position**


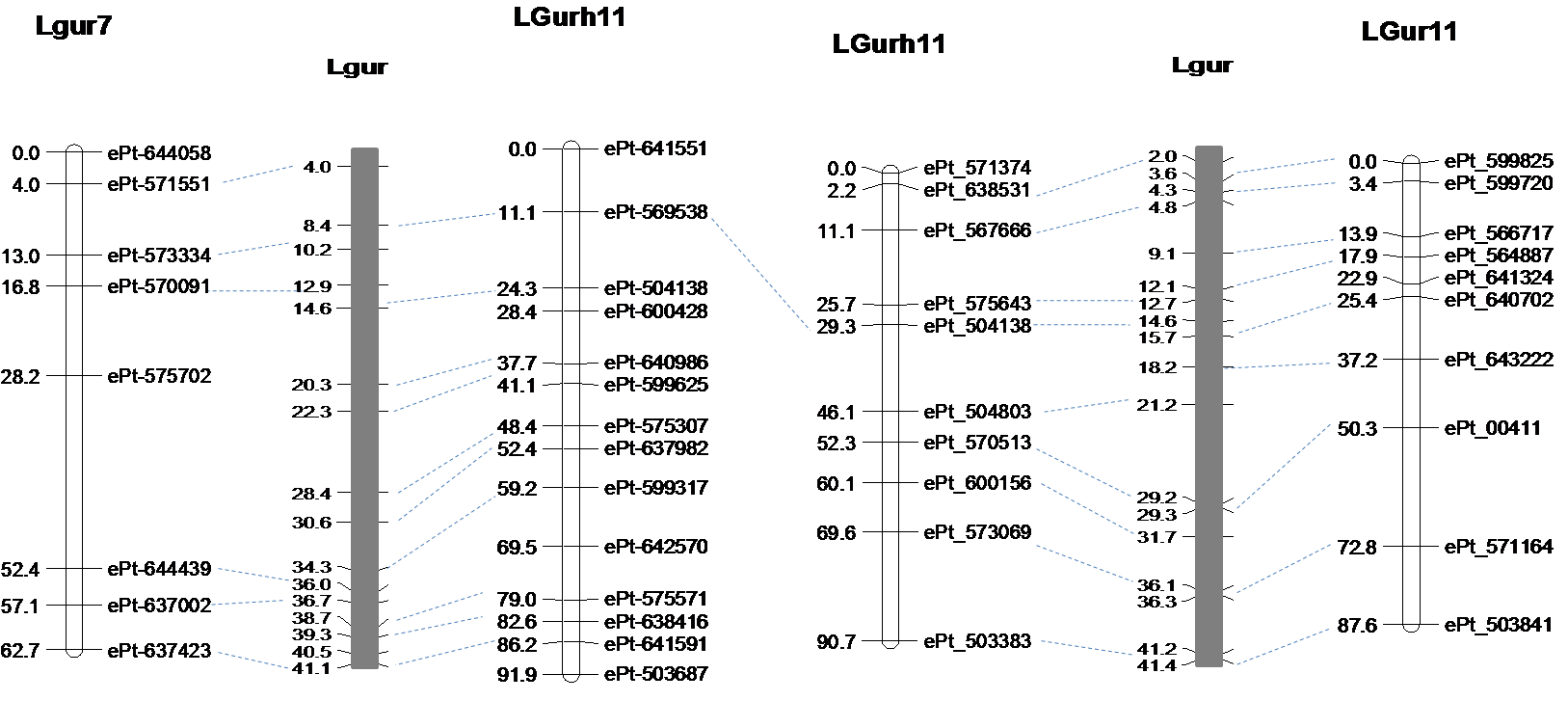

Supplement: Additional file 1 — Table S1. Summary of the framework linkage maps for the E grandis BC family. [file 1471-2156-13-60-S1.doc]
