# Supplementary material for: Genetic dissection of growth, wood basic density and gene expression in interspecific backcrosses of Eucalyptus grandis and E. urophylla
Source: BMC Genet. 2012 Jul 20;13:60. doi: 10.1186/1471-2156-13-60 (PMC3416674; doi:10.1186/1471-2156-13-60)
Supplement: Additional file 2 — Table S2. Summary of the framework linkage maps for the E urophylla BC family. [file 1471-2156-13-60-S2.doc]

**Electronic supplementary material: Supplementary Figure 2**

**Title:** Genetic dissection of growth, wood basic density and gene expression in interspecific backcrosses of *Eucalyptus grandis* and *E. urophylla*

**Journal name :** BMC Genetics

**Authors:** Anand R.K. Kullan, Maria M van Dyk, Charles A. Hefer, Nicoletta Jones, Arnulf Kanzler, Alexander A. Myburg*

**Affiliation and e-mail address of corresponding author:**

Department of Genetics, Forestry and Agricultural Biotechnology Institute (FABI), University of Pretoria, Pretoria, 0002, South Africa

zander.myburg@fabi.up.ac.za

**Supplementary Figure 2**. Epistatic interaction identified by QTLNetwork [74] in the F_1_ hybrid map (*E. urophylla* BC family). The upper panel shows the epistatic interaction detected among three wood basic density QTLs on linkage groups 2, 8 and 10. The red circles represent the positions of QTLs identified by QTLNetwork and the red line connects interacting loci. Only linkage groups with main effects detected are shown. The interaction effects are shown in the lower panel.


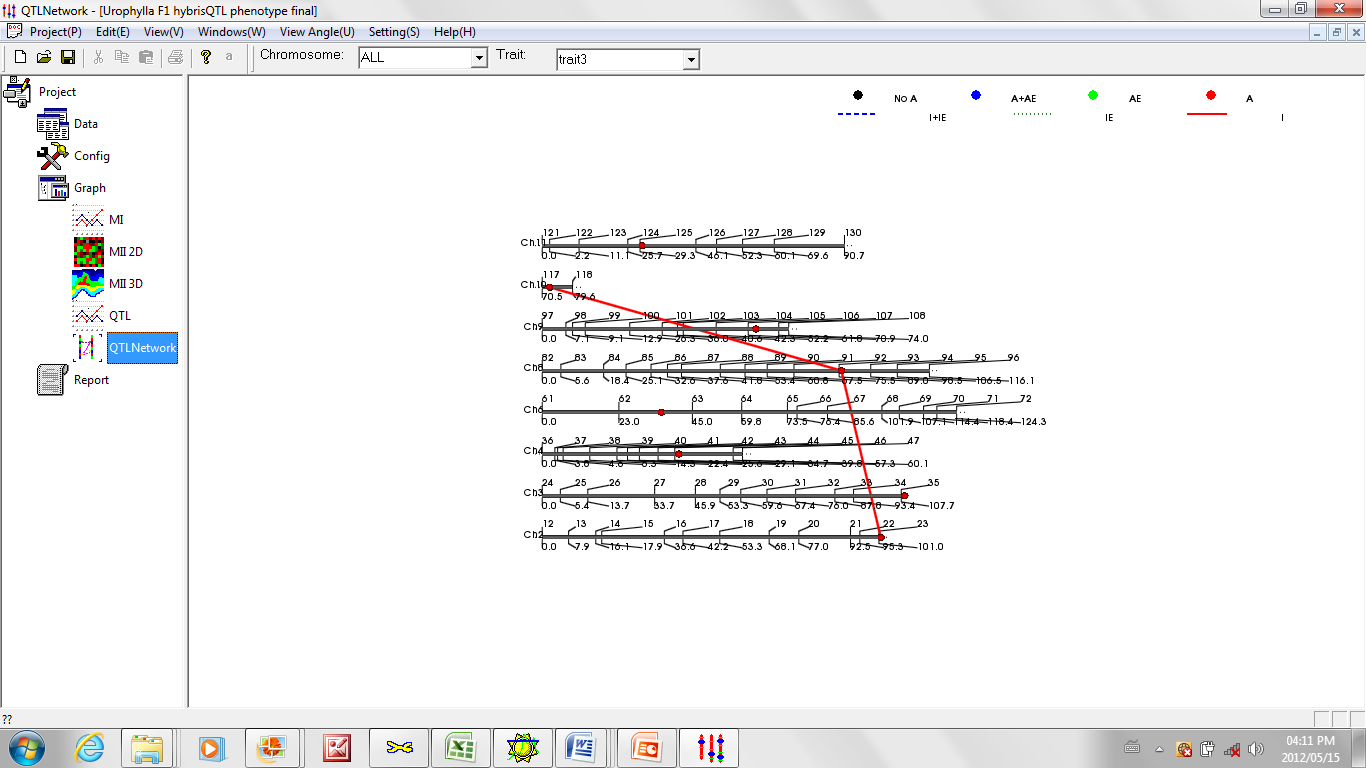


| QTL | Position (cM) | QTL | Position (cM) | AA | SE | *P* value |
| --- | --- | --- | --- | --- | --- | --- |
| LG2 | 100.3 | LG8 | 88.5 | 0.0501 | 0.02 | 0.0000 |
| LG8 | 88.5 | LG10 | 71.5 | -0.0458 | 0.0021 | 0.0000 |

AA, Additive by additive effect: SE, Standard error
