# Supplementary material for: Genetic dissection of growth, wood basic density and gene expression in interspecific backcrosses of Eucalyptus grandis and E. urophylla
Source: BMC Genet. 2012 Jul 20;13:60. doi: 10.1186/1471-2156-13-60 (PMC3416674; doi:10.1186/1471-2156-13-60)
Supplement: Additional file 3 — Figure S1. Comparative QTL mapping of the E. grandis, E. urophylla and their F1 hybrid. [file 1471-2156-13-60-S3.doc]

**Electronic supplementary material: Supplementary Table 3**

**Title:** Genetic dissection of growth, wood basic density and gene expression in interspecific backcrosses of *Eucalyptus grandis* and *E. urophylla*

**Journal name:** BMC Genetics

**Authors:** Anand R.K. Kullan, Maria M van Dyk, Charles A. Hefer, Nicoletta Jones, Arnulf Kanzler, Alexander A. Myburg*

**Affiliation and e-mail address of corresponding author:**

Department of Genetics, Forestry and Agricultural Biotechnology Institute (FABI), University of Pretoria, Pretoria, 0002, South Africa

zander.myburg@fabi.up.ac.za

**Supplementary Table 3a. eQTLs identified for the top 10 most positively correlated genes (transcript abundance correlated with wood basic density) under the epistatic QTL region on LG8 of the F_1_ hybrid (*E. urophylla* BC family). A total of three and eight genes share trans-eQTLs on LG4 (10-22 cM) and LG10 (52-88 cM) that correspond to wood basic density QTLs on the same linkage groups. A putative trans-eQTL was also detected for six genes on LG6 (85-120 cM), but no wood density QTL was detected in this region.**

| **Gene identification** | **Physical position (bp)** | **At. identification** | **At. description** | **eQTL on linkage group** | **eQTL position (cM)** | **Correlation with wood density (R^2^)** |
| --- | --- | --- | --- | --- | --- | --- |
| Eucgr.H03646.1 | 53,552,147 | AT3G21550.1 | DUF679 domain membrane protein 2 | 6, 10 | 85, 54 | 0.54 |
| Eucgr.H03652.1 | 53613563 | AT5G18910.1 | Protein kinase superfamily protein | 6, 10 | 101, 52 | 0.52 |
| Eucgr.H04339.1 | 62373141 | AT5G28840.1 | GDP-D-mannose 3',5'-epimerase | 4, 6, 10 | 14, 101, 54 | 0.52 |
| Eucgr.H04134.1 | 59247365 | AT1G62290.1 | Saposin-like aspartyl protease family protein | 6, 10 | 120, 70 | 0.51 |
| Eucgr.H04474.1 | 64015570 | AT5G19090.1 | Heavy metal transport/detoxification superfamily protein | 4, 6, 10 | 10, 93, 52 | 0.49 |
| Eucgr.H04133.1 | 59246267 | AT4G04460.1 | Saposin-like aspartyl protease family protein | 4, 6, 10 | 22, 99, 54 | 0.48 |
| Eucgr.H04329.1 | 62264300 | AT3G04720.1 | pathogenesis-related 4 | - | - | 0.46 |
| Eucgr.H03914.1 | 56889409 | AT5G13930.1 | Chalcone and stilbene synthase family protein | 10 | 72 | 0.45 |
| Eucgr.H03673.1 | 53833668 |  | Unknown | 8 | 65 | 0.43 |
| Eucgr.H04139.1 | 59277899 | AT1G48320.1 | Thioesterase superfamily protein | 7, 10 | 46, 88 | 0.42 |
| At., *Arabidopsis thaliana*; eQTL, Expression quantitative trait locus; Eucgr., *Eucalyptus grandis*; QTL, Quantitative trait locus. | | | | | | |

**Supplementary Table 3b: eQTLs identified for the top 10 genes (whose transcript abundance were negatively correlated with wood density trait variation) located in the wood basic density QTL intervals on LG8 and LG9 (F_1_ hybrid map, *E. urophylla* backcross family).**

| **Gene identification** | **Linkage group (LG)** | **Physical position (bp)** | **At. identification** | **At. description** | **eQTL on linkage group** | **eQTL position (cM)** | **Correlation with wood density (R^2^)** |
| --- | --- | --- | --- | --- | --- | --- | --- |
| Eucgr.H03971.1 | LG8 | 57716920 | AT1G06330.1 | Heavy metal transport/detoxification superfamily protein | 4, 8 | 39, 71 | 0.42 |
| Eucgr.H04207.1 | LG8 | 60095955 | AT5G18460.1 | Protein of Unknown Function (DUF239) | 9 | 2.0 | 0.40 |
| Eucgr.H03952.1 | LG8 | 57402958 | AT5G49130.1 | MATE efflux family protein | 10 | 72 | 0.35 |
| Eucgr.H04141.1 | LG8 | 59329525 | AT3G17600.1 | indole-3-acetic acid inducible 31 | 10 | 72 | 0.33 |
| Eucgr.H04476.1 | LG8 | 64030893 | AT1G08970.1 | nuclear factor Y, subunit C9 | 4, 8, 10 | 18, 90, 73 | 0.33 |
| Eucgr.H04042.1 | LG8 | 58287114 | AT3G17040.2 | high chlorophyll fluorescent 107 | 2, 8 | 74, 77 | 0.33 |
| Eucgr.H04058.1 | LG8 | 58443103 | - | Unknown | 4, 8 | 27, 63 | 0.32 |
| Eucgr.H04118.1 | LG8 | 59125445 | AT1G56720.1 | Protein kinase superfamily protein | 8 | 88 | 0.32 |
| Eucgr.H04336.1 | LG8 | 62350663 | AT5G43700.1 | AUX/IAA transcriptional regulator family protein | 5, 8 | 44, 88 | 0.30 |
| Eucgr.H04045.1 | LG8 | 58315064 | AT5G49460.1 | ATP citrate lyase subunit B 2 | 10 | 51 | 0.30 |
| Eucgr.I02511.1 | LG9 | 36197592 | AT2G14520.1 | CBS domain-containing protein with a domain of unknown function (DUF21) | 9 | 70 | 0.34 |
| Eucgr.I02585.1 | LG9 | 37144011 | AT5G64410.1 | oligopeptide transporter 4 | 8, 9, 10 | 88, 70, 54 | 0.32 |
| Eucgr.I02426.1 | LG9 | 34863154 | AT2G18260.1 | syntaxin of plants 112 | - | - | 0.31 |
| Eucgr.I02587.1 | LG9 | 37175082 | AT5G64410.1 | oligopeptide transporter 4 | 9 | 70 | 0.30 |
| Eucgr.I01897.1 | LG9 | 28902234 | AT3G28480.1 | Oxoglutarate/iron-dependent oxygenase | 9 | 58 | 0.30 |
| Eucgr.I02119.1 | LG9 | 30935715 | AT5G15900.1 | TRICHOME BIREFRINGENCE-LIKE 19 | 9 | 61 | 0.29 |
| Eucgr.I02091.1 | LG9 | 30613087 | AT3G02350.1 | galacturonosyltransferase 9 | - | - | 0.29 |
| Eucgr.I01898.1 | LG9 | 28908553 | AT5G40020.1 | Pathogenesis-related thaumatin superfamily protein | 9 | 52 | 0.29 |
| Eucgr.I02197.1 | LG9 | 31874363 | AT1G76520.1 | Auxin efflux carrier family protein | 9 | 60 | 0.27 |
| Eucgr.I02308.1 | LG9 | 33653685 | AT4G35840.1 | RING/U-box superfamily protein | 10 | 72 | 0.27 |
| At., *Arabidopsis thaliana*; eQTL, Expression quantitative trait locus; Eucgr., *Eucalyptus grandis*; QTL, Quantitative trait locus. | | | | | | | |
