# Supplementary material for: Genetic dissection of growth, wood basic density and gene expression in interspecific backcrosses of Eucalyptus grandis and E. urophylla
Source: BMC Genet. 2012 Jul 20;13:60. doi: 10.1186/1471-2156-13-60 (PMC3416674; doi:10.1186/1471-2156-13-60)
Supplement: Additional file 4 — Figure S2. Epistatic interaction identified between LG8 and 10. [file 1471-2156-13-60-S4.doc]

**Electronic supplementary material: Supplementary Table 4**

**Title:** Genetic dissection of growth, wood basic density and gene expression in interspecific backcrosses of *Eucalyptus grandis* and *E. urophylla*

**Journal name:** BMC Genetics

**Authors:** Anand R.K. Kullan, Maria M van Dyk, Charles A. Hefer, Nicoletta Jones, Arnulf Kanzler, Alexander A. Myburg*

**Affiliation and e-mail address of corresponding author:**

Department of Genetics, Forestry and Agricultural Biotechnology Institute (FABI), University of Pretoria, Pretoria, 0002, South Africa

zander.myburg@fabi.up.ac.za

**Supplementary Table 4. QTL studies reported for DBH and wood density in *Eucalyptus***

| **Cross** | **Number of progeny** | **Marker used** | **Trait name** | **Number of QTLs identified** | **Percentage of variation explained by the QTLs** | **Reference** |
| --- | --- | --- | --- | --- | --- | --- |
| *E. grandis* x  *E. urophylla*  *E. urophylla x E. grandis*  *E. globulus* x *E. globulus*  *E. globulus* x *E. globulus*  *E. globulus x E. globulus*  *E. nitens x E. nitens*  *E. urophylla x E. grandis* | 1085  201  117  135-148  112  327  201 | RAPD  RAPD  RAPD and microsatellites  RFLP and microsatellites  AFLP and microsatellites  RFLP and microsatellites  RAPD, SSCP and microsatellites | Volume growth  Wood density  Tree diameter  Wood density  Cumulative growth  Wood density  Wood density  Tree diameter  Wood density  Tree diameter  Wood density  Tree diameter  Wood density | 3  5  3  1 to 4  1  2  4  2  3  1 to 3  5  1 to 3  2 to 4 | 3.9 to 6.6 %  3.4 to 10.2 %  5.1 to 14%  5.1 to 11.0 %  8.0 to 17.9  3.2 to 15.8%  3.8 to 5.2 %  7.2 to 10.4 %  3.3 to 7. 2%  2.8 to 6.9 %  5.1 to 11.9%  4.4 to 25.4% | Grattapaglia et al. 1996  Verhaegen *et al*. 1997  Bundock et al. 2008  Thamarus et al. 2004  Freeman et al. 2009  Thumma et al. 2010  Gion et al. 2011 |
