# Supplementary material for: Genetic dissection of growth, wood basic density and gene expression in interspecific backcrosses of Eucalyptus grandis and E. urophylla
Source: BMC Genet. 2012 Jul 20;13:60. doi: 10.1186/1471-2156-13-60 (PMC3416674; doi:10.1186/1471-2156-13-60)
Supplement: Additional file 6 — Figure S3. eQTLs for the genes underlying a major wood density QTL. [file 1471-2156-13-60-S6.doc]

**Electronic supplementary material: Supplementary Table 5**

**Title:** Genetic dissection of growth, wood basic density and gene expression in interspecific backcrosses of *Eucalyptus grandis* and *E. urophylla*

**Journal name:** BMC Genetics

**Authors:** Anand R.K. Kullan, Maria M van Dyk, Charles A. Hefer, Nicoletta Jones, Arnulf Kanzler, Alexander A. Myburg

**Affiliation and e-mail address of corresponding author:**

Department of Genetics, Forestry and Agricultural Biotechnology Institute (FABI), University of Pretoria, Pretoria, 0002, South Africa

zander.myburg@fabi.up.ac.za

**Supplementary Table 5.** Comparison of linkage group assignment of QTLs for DBH detected in this study and previous QTL mapping studies in *Eucalyptus*

| **Linkage group** | **This study** | | | | **Bundock**  **et al. (2008)** | **Freeman**  **et al. (2009)** | **Thumma**  **et al. (2010a, b)** |  |
| --- | --- | --- | --- | --- | --- | --- | --- | --- |
|  | ***E. grandis*** | **F1 hybrid**  ***(E. grandis* BC family)** | ***E. urophyll*a** | **F1 hybrid**  **(*E. urophylla* BC family)** | ***E. globulus*** | ***E. globulus*** | ***E. nitens*** | **Total** |
| LG1 |  |  |  |  |  |  |  | **-** |
| LG2 |  |  |  |  |  |  |  |  |
| LG3 |  |  |  |  |  |  |  | **-** |
| LG4 |  |  |  |  |  |  |  | **-** |
| LG5 |  |  |  |  | 1 | 1 | 1 | **3** |
| LG6 |  | 1 |  | 1 |  |  | 1 | **3** |
| LG7 |  |  |  |  |  |  |  | **-** |
| LG8 |  |  |  |  |  |  | 1 | **1** |
| LG9 | 1 |  |  |  |  |  |  | **1** |
| LG10 |  | 1 | 1 |  |  | 1 |  | **3** |
| LG11 |  |  |  |  |  |  |  | **-** |
| **Total** | **1** | **2** | **1** | **1** | **1** | **2** | **3** | **11** |
