# Supplementary material for: Genetic dissection of growth, wood basic density and gene expression in interspecific backcrosses of Eucalyptus grandis and E. urophylla
Source: BMC Genet. 2012 Jul 20;13:60. doi: 10.1186/1471-2156-13-60 (PMC3416674; doi:10.1186/1471-2156-13-60)

**Electronic supplementary material: Supplementary Figure 3**

**Title:** Genetic dissection of growth, wood basic density and gene expression in interspecific backcrosses of *Eucalyptus grandis* and *E. urophylla*

**Journal name:** BMC Genetics

**Authors:** Anand R.K. Kullan, Maria M van Dyk, Charles A. Hefer, Nicoletta Jones, Arnulf Kanzler, Alexander A. Myburg

**Affiliation and e-mail address of corresponding author:**

Department of Genetics, Forestry and Agricultural Biotechnology Institute (FABI), University of Pretoria, Pretoria, 0002, South Africa

zander.myburg@fabi.up.ac.za

**Supplementary Figure 3**. **Genome-wide positions of eQTLs detected for genes underlying a major wood basic density QTL interval on linkage group 9 of the F_1_ hybrid (*E. urophylla* backcross family)**. Frequency of eQTLs (cM, Kosambi) identified for 294 genes underlying the wood density QTL interval (black horizontal bar) on linkage group 9 (Table 3, Figure 2). eQTLs that overlap the wood density interval (and the positions of the 294 genes in the interval) are classified as cis-eQTLs and the rest as trans-eQTLs.


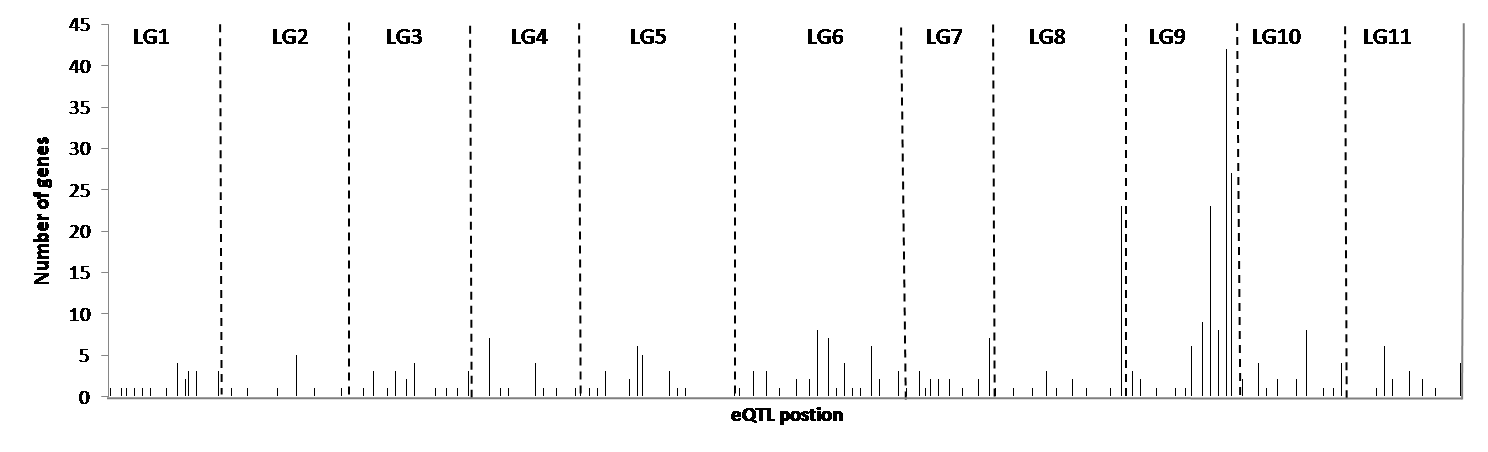

Supplement: Additional file 7 — Table S3. eQTLs identified for genes on LG8 and LG9. [file 1471-2156-13-60-S7.doc]
