# Supplementary material for: Genetic dissection of growth, wood basic density and gene expression in interspecific backcrosses of Eucalyptus grandis and E. urophylla
Source: BMC Genet. 2012 Jul 20;13:60. doi: 10.1186/1471-2156-13-60 (PMC3416674; doi:10.1186/1471-2156-13-60)
Supplement: Additional file 9 — Table S4. QTLs studies reported for DBH and wood density in Eucalyptus. [file 1471-2156-13-60-S9.doc]

**Electronic supplementary material: Supplementary Figure 4**

**Title:** Genetic dissection of growth, wood basic density and gene expression in interspecific backcrosses of *Eucalyptus grandis* and *E. urophylla*

**Journal name:** BMC Genetics

**Authors:** Anand R.K. Kullan, Maria M van Dyk, Charles A. Hefer, Nicoletta Jones, Arnulf Kanzler, Alexander A. Myburg*

**Affiliation and e-mail address of corresponding author:**

Department of Genetics, Forestry and Agricultural Biotechnology Institute (FABI), University of Pretoria, Pretoria, 0002, South Africa

zander.myburg@fabi.up.ac.za

**Supplementary Figure 4. Schematic representation of the segregation of dominant effect QTLs from the F_1_ hybrid in the two backcross families.**  Under complete dominance, in case of an F_1_ hybrid heterozygous for a dominant allele (Qq) at a trait locus backcrossed with homozygous Parent 1 (QQ), the resulting backcross progeny would all carry at least one copy of the dominant Q allele, so there would not be any segregation for the trait locus in the backcross progeny and a QTL would not be detected. On the other hand, if the F_1_ hybrid (Qq) was backcrossed to a recessive Parent 2 (qq), the resulting backcross progeny would either be heterozygous (Qq) or homozygous (qq) at this locus (1:1 segregation ratio), enabling the identification of a QTL.

**QQ**

**Qq**

**qq**

x

x

No phenotypic segregation under complete dominance

Segregation (1:1)

(F_1_ hybrid parent)

(BC Parent 2)

(BC Parent 1)
