# Supplementary material for: Genetic dissection of growth, wood basic density and gene expression in interspecific backcrosses of Eucalyptus grandis and E. urophylla
Source: BMC Genet. 2012 Jul 20;13:60. doi: 10.1186/1471-2156-13-60 (PMC3416674; doi:10.1186/1471-2156-13-60)
Supplement: Additional file 11 — Table S6. Comparative QTLs for wood density among different Eucalyptus species. [file 1471-2156-13-60-S11.doc]

**Electronic supplementary material: Supplementary Table 2**

**Title:** Genetic dissection of growth, wood basic density and gene expression in interspecific backcrosses of *Eucalyptus grandis* and *E. urophylla*

**Journal name :** BMC Genetics

**Authors:** Anand R.K. Kullan, Maria M van Dyk, Charles A. Hefer, Nicoletta Jones, Arnulf Kanzler, Alexander A. Myburg*

**Affiliation and e-mail address of corresponding author:**

Department of Genetics, Forestry and Agricultural Biotechnology Institute (FABI), University of Pretoria, Pretoria, 0002, South Africa

zander.myburg@fabi.up.ac.za

***Supplementary Table 2a.*** *Summary of the framework linkage maps constructed for QTL analysis in the E. urophylla backcross family.*

|  | ***E. urophylla* backcross parent map** | | | **F_1_ hybrid parent map (*E. urophylla* backcross family)** | | |
| --- | --- | --- | --- | --- | --- | --- |
| **Linkage group** | **Number of markers** | **Linkage group length (cM)** | **Mean distance between markers (cM)** | **Number of markers** | **Linkage group length (cM)** | **Mean distance between markers (cM)** |
| 1  2  3  4  5  6  7  8  9  10  11 | 8  8  13  8  10  14  10  13  8  10  10 | 82.4  38.6  100.4  72.0  88.9  100.6  79.5  112.4  95.4  97.1  87.6 | 10.3  4.8  7.7  9.0  8.8  7.1  7.9  8.6  11.9  9.7  8.7 | 11  12  12  12  13  14  9  13  12  12  10 | 91.3  101.0  107.7  60.1  95.2  124.3  74.0  108.4  74.0  91.9  90.7 | 8.3  8.4  8.9  5.0  7.3  8.8  8.2  8.3  6.1  7.6  9.0 |
| **Total** | **112** | **954.9** | **8.5** | **130** | **1018.0** | **7.8** |
| BC, backcross; QTL, Quantitative trait locus. | | | | | | |

**Supplementary Table 2b. Markers used for QTL analysis, their positions and additive effects in the *E. urophylla* backcross family.**

| ***E. urophylla* backcross parent** | | | | | **F1 hybrid of *E. urophylla* BC family** | | | | |
| --- | --- | --- | --- | --- | --- | --- | --- | --- | --- |
| **Marker Name** | **Linkage group (LG)** | **Map Position (cM)** | **Additive effect (SD, DBH)** | **Additive effect (SD, Density)** | **Marker Name** | **Linkage group (LG)** | **Map Position (cM)** | **Additive effect (SD, DBH)^a^** | **Additive effect (SD, Density)^a^** |
| ePt_638907 | LG1 | 0.00 | 0.05 | 0.06 | ePt_503701 | LG1 | 0.00 | 0.02 | 0.01 |
| ePt_640386 | LG1 | 2.73 | 0.06 | 0.04 | ePt_503224 | LG1 | 10.54 | 0.01 | 0.00 |
| ePt_571510 | LG1 | 16.49 | 0.08 | 0.06 | ePt_573788 | LG1 | 17.87 | 0.06 | 0.05 |
| ePt_636534 | LG1 | 46.12 | 0.13 | 0.03 | ePt_572856 | LG1 | 30.50 | -0.04 | -0.10 |
| ePt_571936 | LG1 | 50.44 | 0.17 | 0.00 | ePt_600232 | LG1 | 36.17 | 0.05 | -0.07 |
| ePt_568625 | LG1 | 70.49 | 0.12 | 0.07 | ePt_642824 | LG1 | 46.92 | 0.22 | -0.03 |
| ePt_641800 | LG1 | 78.78 | 0.15 | 0.05 | ePt_599740 | LG1 | 56.39 | 0.16 | -0.11 |
| ePt_636825 | LG1 | 82.36 | 0.14 | 0.03 | ePt_565915 | LG1 | 65.47 | 0.30 | -0.06 |
| ePt_637636 | LG2 | 0.00 | 0.05 | 0.06 | ePt_600664 | LG1 | 81.49 | -0.10 | -0.06 |
| ePt_563419 | LG2 | 4.62 | 0.07 | 0.08 | ePt_639849 | LG1 | 87.91 | 0.02 | -0.06 |
| ePt_565691 | LG2 | 14.27 | 0.14 | 0.12 | ePt_563014 | LG1 | 91.34 | 0.01 | -0.06 |
| ePt_574308 | LG2 | 22.37 | 0.12 | 0.06 | ePt_505021 | LG2 | 0.00 | 0.19 | -0.15 |
| ePt_637223 | LG2 | 26.94 | 0.23 | 0.11 | ePt_574374 | LG2 | 7.92 | 0.13 | -0.04 |
| ePt_575592 | LG2 | 29.16 | 0.27 | 0.09 | ePt_567712 | LG2 | 16.07 | 0.09 | -0.04 |
| ePt_575031 | LG2 | 36.01 | 0.21 | 0.14 | ePt_569091 | LG2 | 17.86 | 0.09 | -0.05 |
| ePt_566116 | LG2 | 38.56 | 0.20 | 0.12 | ePt_639347 | LG2 | 36.63 | -0.14 | -0.02 |
| ePt_564331 | LG3 | 0.00 | 0.08 | 0.05 | ePt_504902 | LG2 | 42.22 | -0.09 | -0.02 |
| ePt_503896 | LG3 | 4.05 | 0.03 | 0.06 | ePt_573048 | LG2 | 53.26 | -0.10 | -0.12 |
| ePt_599238 | LG3 | 9.08 | 0.10 | 0.07 | ePt_568036 | LG2 | 68.07 | -0.14 | -0.07 |
| ePt_566346 | LG3 | 15.38 | 0.11 | 0.03 | ePt_567298 | LG2 | 76.97 | -0.03 | -0.22 |
| ePt_643823 | LG3 | 20.67 | 0.12 | 0.03 | ePt_503852 | LG2 | 92.48 | 0.09 | -0.33 |
| ePt_641822 | LG3 | 26.72 | 0.22 | 0.02 | ePt_565848 | LG2 | 95.27 | 0.07 | -0.30 |
| ePt_504319 | LG3 | 38.47 | 0.23 | 0.07 | ePt_641866 | LG2 | 101.00 | 0.02 | -0.39 |
| ePt_637193 | LG3 | 45.45 | 0.11 | 0.11 | ePt_644293 | LG3 | 0.00 | 0.12 | -0.13 |
| ePt_572258 | LG3 | 59.13 | 0.03 | 0.06 | ePt_568037 | LG3 | 5.44 | 0.04 | -0.07 |
| ePt_644491 | LG3 | 70.44 | 0.08 | 0.20 | ePt_572721 | LG3 | 13.68 | 0.07 | -0.12 |
| ePt_642546 | LG3 | 79.28 | 0.08 | 0.19 | ePt_568116 | LG3 | 33.73 | -0.04 | 0.07 |
| ePt_600566 | LG3 | 92.96 | 0.01 | 0.20 | ePt_644403 | LG3 | 45.94 | -0.03 | 0.03 |
| ePt_566961 | LG3 | 100.42 | 0.03 | 0.17 | ePt_640977 | LG3 | 53.33 | -0.02 | -0.08 |
| ePt_567857 | LG4 | 0.00 | 0.35 | 0.10 | ePt_640786 | LG3 | 59.57 | 0.02 | -0.13 |
| ePt_567945 | LG4 | 3.07 | 0.29 | 0.16 | ePt_567607 | LG3 | 67.42 | 0.13 | -0.31 |
| ePt_569322 | LG4 | 7.23 | 0.25 | 0.19 | ePt_571789 | LG3 | 76.02 | 0.17 | -0.35 |
| ePt_642860 | LG4 | 30.64 | 0.07 | 0.12 | ePt_566415 | LG3 | 87.80 | 0.23 | -0.36 |
| ePt_637285 | LG4 | 37.23 | 0.11 | 0.02 | ePt_636665 | LG3 | 93.39 | 0.17 | -0.33 |
| ePt_599283 | LG4 | 58.90 | 0.05 | 0.21 | ePt_570151 | LG3 | 107.70 | 0.15 | -0.38 |
| ePt_570780 | LG4 | 65.23 | 0.06 | 0.24 | ePt_570493 | LG4 | 0.00 | 0.07 | 0.04 |
| ePt_636880 | LG4 | 72.03 | 0.07 | 0.19 | ePt_600655 | LG4 | 3.79 | 0.10 | 0.04 |
| ePt_643707 | LG5 | 0.00 | 0.08 | 0.12 | ePt_503766 | LG4 | 4.62 | 0.11 | 0.01 |
| ePt_639138 | LG5 | 6.23 | 0.15 | 0.14 | ePt_643984 | LG4 | 6.27 | 0.12 | 0.10 |
| ePt_571353 | LG5 | 18.81 | 0.01 | 0.04 | ePt_636515 | LG4 | 14.28 | 0.18 | 0.06 |
| ePt_572057 | LG5 | 35.54 | 0.05 | 0.02 | ePt_571769 | LG4 | 22.42 | 0.19 | -0.01 |
| ePt_636498 | LG5 | 48.88 | 0.08 | 0.05 | ePt_573193 | LG4 | 25.58 | 0.19 | -0.06 |
| ePt_573321 | LG5 | 55.79 | 0.01 | 0.06 | ePt_599702 | LG4 | 29.14 | 0.18 | 0.51 |
| ePt_636480 | LG5 | 66.81 | 0.01 | 0.15 | ePt_573837 | LG4 | 34.75 | 0.15 | 0.56 |
| ePt_643842 | LG5 | 78.12 | 0.03 | 0.12 | ePt_638305 | LG4 | 39.77 | 0.19 | 0.62 |
| ePt_600653 | LG5 | 85.68 | 0.08 | 0.18 | ePt_503674 | LG4 | 57.27 | 0.16 | -0.17 |
| ePt_503808 | LG5 | 88.87 | 0.07 | 0.20 | ePt_570682 | LG4 | 60.09 | 0.19 | -0.13 |
| ePt_568290 | LG6 | 0.00 | 0.03 | 0.17 | ePt_570054 | LG5 | 0.00 | -0.03 | 0.04 |
| ePt_575429 | LG6 | 1.12 | 0.04 | 0.16 | ePt_643259 | LG5 | 9.46 | 0.09 | 0.01 |
| ePt_636902 | LG6 | 5.86 | 0.07 | 0.11 | ePt_567769 | LG5 | 17.14 | 0.08 | -0.05 |
| ePt_505086 | LG6 | 14.44 | 0.02 | 0.01 | ePt_644484 | LG5 | 19.99 | 0.07 | -0.06 |
| ePt_636956 | LG6 | 17.66 | 0.02 | 0.05 | ePt_570274 | LG5 | 31.29 | 0.08 | -0.12 |
| ePt_644163 | LG6 | 31.49 | 0.27 | 0.04 | ePt_575685 | LG5 | 36.70 | 0.07 | -0.11 |
| ePt_638183 | LG6 | 43.44 | 0.24 | 0.06 | ePt_639908 | LG5 | 45.52 | 0.15 | -0.04 |
| ePt_637303 | LG6 | 50.76 | 0.28 | 0.10 | ePt_569419 | LG5 | 50.88 | 0.21 | -0.05 |
| ePt_569463 | LG6 | 63.45 | 0.08 | 0.02 | ePt_568347 | LG5 | 57.71 | 0.15 | -0.01 |
| ePt_641907 | LG6 | 67.82 | 0.16 | 0.02 | ePt_575678 | LG5 | 64.98 | 0.11 | 0.02 |
| ePt_639634 | LG6 | 82.84 | 0.14 | 0.16 | ePt_574584 | LG5 | 78.05 | 0.09 | 0.04 |
| ePt_640477 | LG6 | 89.20 | 0.21 | 0.16 | ePt_573934 | LG5 | 80.80 | 0.11 | 0.00 |
| ePt_565994 | LG6 | 103.14 | 0.23 | 0.10 | ePt_643170 | LG5 | 95.23 | 0.04 | 0.01 |
| ePt_566534 | LG6 | 110.56 | 0.28 | 0.10 | ePt_565718 | LG6 | 0.00 | -0.17 | -0.37 |
| ePt_566646 | LG7 | 0.00 | 0.06 | 0.05 | ePt_641950 | LG6 | 23.00 | -0.19 | -0.47 |
| ePt_571137 | LG7 | 2.67 | 0.07 | 0.20 | ePt_563588 | LG6 | 27.73 | 0.18 | -0.44 |
| ePt_573687 | LG7 | 10.17 | 0.12 | 0.29 | ePt_599939 | LG6 | 37.06 | 0.36 | -0.03 |
| ePt_637829 | LG7 | 10.61 | 0.11 | 0.31 | ePt_504039 | LG6 | 45.05 | 0.47 | -0.15 |
| ePt_563468 | LG7 | 19.98 | 0.11 | 0.37 | ePt_566935 | LG6 | 59.85 | 0.25 | -0.20 |
| ePt_637575 | LG7 | 35.70 | 0.16 | 0.04 | ePt_641192 | LG6 | 73.55 | 0.09 | -0.18 |
| ePt_566192 | LG7 | 62.52 | 0.05 | 0.15 | ePt_643704 | LG6 | 76.43 | 0.10 | -0.19 |
| ePt_571262 | LG7 | 70.61 | 0.06 | 0.18 | ePt_504218 | LG6 | 85.57 | -0.17 | -0.32 |
| ePt_644456 | LG7 | 72.78 | 0.09 | 0.19 | ePt_643314 | LG6 | 101.90 | -0.10 | -0.21 |
| ePt_566482 | LG7 | 79.47 | 0.03 | 0.27 | ePt_575722 | LG6 | 107.12 | -0.22 | -0.18 |
| ePt_639224 | LG8 | 0.00 | 0.09 | 0.07 | ePt_638034 | LG6 | 114.40 | 0.12 | -0.07 |
| ePt_504097 | LG8 | 8.17 | 0.04 | 0.01 | ePt_568058 | LG6 | 118.37 | -0.28 | -0.02 |
| ePt_565436 | LG8 | 11.79 | 0.04 | 0.03 | ePt_574570 | LG6 | 124.32 | -0.18 | -0.01 |
| ePt_575292 | LG8 | 12.50 | 0.05 | 0.19 | ePt_642265 | LG7 | 0.00 | -0.23 | -0.14 |
| ePt_574247 | LG8 | 36.24 | 0.04 | 0.37 | ePt_568515 | LG7 | 5.52 | -0.23 | -0.06 |
| ePt_639354 | LG8 | 44.53 | 0.02 | 0.35 | ePt_571000 | LG7 | 23.29 | -0.14 | 0.12 |
| ePt_637292 | LG8 | 53.21 | 0.07 | 0.00 | ePt_575149 | LG7 | 34.99 | -0.07 | 0.01 |
| ePt_564161 | LG8 | 67.69 | 0.01 | 0.01 | ePt_643010 | LG7 | 43.49 | -0.10 | -0.04 |
| ePt_503998 | LG8 | 73.25 | 0.01 | 0.02 | ePt_574160 | LG7 | 46.16 | -0.07 | -0.02 |
| ePt_637028 | LG8 | 90.80 | 0.12 | 0.07 | ePt_639371 | LG7 | 56.16 | -0.09 | -0.05 |
| ePt_574691 | LG8 | 93.35 | 0.13 | 0.07 | ePt_568436 | LG7 | 64.60 | -0.11 | -0.08 |
| ePt_570192 | LG8 | 107.51 | 0.11 | 0.00 | ePt_564451 | LG7 | 73.97 | -0.08 | -0.03 |
| ePt_644171 | LG8 | 112.35 | 0.17 | 0.06 | ePt_504398 | LG8 | 0.00 | 0.05 | 0.26 |
| ePt_572414 | LG9 | 0.00 | 0.17 | 0.05 | ePt_571985 | LG8 | 11.03 | 0.09 | 0.21 |
| ePt_571185 | LG9 | 13.53 | 0.13 | 0.13 | ePt_565753 | LG8 | 16.28 | 0.16 | 0.14 |
| ePt_571912 | LG9 | 25.42 | 0.26 | 0.16 | ePt_599669 | LG8 | 20.98 | 0.17 | 0.05 |
| ePt_566194 | LG9 | 30.85 | 0.40 | 0.20 | ePt_572210 | LG8 | 29.04 | 0.15 | 0.03 |
| ePt_563442 | LG9 | 46.07 | 0.16 | 0.21 | ePt_643850 | LG8 | 48.00 | 0.04 | -0.27 |
| ePt_642787 | LG9 | 72.00 | 0.04 | 0.04 | ePt_503845 | LG8 | 53.61 | -0.01 | -0.33 |
| ePt_573318 | LG9 | 74.05 | 0.04 | 0.08 | ePt_640365 | LG8 | 59.81 | 0.03 | -0.38 |
| ePt_599981 | LG9 | 95.36 | 0.13 | 0.13 | ePt_640629 | LG8 | 67.70 | 0.06 | -0.33 |
| ePt_641100 | LG10 | 0.00 | 0.40 | 0.16 | ePt_575022 | LG8 | 79.29 | 0.16 | -0.37 |
| ePt_570364 | LG10 | 3.51 | 0.43 | 0.21 | ePt_640149 | LG8 | 88.85 | 0.20 | -0.27 |
| ePt_640576 | LG10 | 20.93 | 0.06 | 0.12 | ePt_572120 | LG8 | 97.61 | 0.22 | -0.22 |
| ePt_575378 | LG10 | 32.04 | 0.03 | 0.12 | ePt_573365 | LG8 | 108.47 | 0.10 | -0.22 |
| ePt_568620 | LG10 | 46.40 | 0.03 | 0.09 | ePt_575684 | LG9 | 0.00 | -0.04 | -0.27 |
| ePt_644096 | LG10 | 61.93 | 0.04 | 0.05 | ePt_641785 | LG9 | 7.11 | 0.05 | -0.21 |
| ePt_563364 | LG10 | 73.27 | 0.03 | 0.14 | ePt_567963 | LG9 | 9.11 | 0.07 | -0.18 |
| ePt_642097 | LG10 | 83.94 | 0.22 | 0.19 | ePt_572213 | LG9 | 12.91 | 0.13 | -0.18 |
| ePt_643528 | LG10 | 88.39 | 0.18 | 0.22 | ePt_567473 | LG9 | 26.26 | 0.23 | 0.15 |
| ePt_638623 | LG10 | 97.06 | 0.18 | 0.23 | ePt_644133 | LG9 | 35.99 | 0.19 | 0.34 |
| ePt_599825 | LG11 | 0.00 | 0.04 | 0.02 | ePt_639625 | LG9 | 40.55 | 0.11 | 0.42 |
| ePt_599720 | LG11 | 3.36 | 0.06 | 0.10 | ePt_640713 | LG9 | 42.30 | 0.12 | 0.45 |
| ePt_566717 | LG11 | 13.92 | 0.04 | 0.11 | ePt_599692 | LG9 | 52.17 | 0.09 | 0.59 |
| ePt_564887 | LG11 | 17.94 | 0.01 | 0.14 | ePt_600528 | LG9 | 61.79 | 0.04 | 0.68 |
| ePt_641324 | LG11 | 22.85 | 0.02 | 0.13 | ePt_639912 | LG9 | 70.95 | 0.12 | 0.64 |
| ePt_640702 | LG11 | 25.43 | 0.07 | 0.09 | ePt_504705 | LG9 | 73.95 | 0.09 | 0.65 |
| ePt_643222 | LG11 | 37.19 | 0.07 | 0.21 | ePt_565743 | LG10 | 0.00 | 0.12 | 0.42 |
| ePt_00411 | LG11 | 50.32 | 0.10 | 0.25 | ePt_637643 | LG10 | 6.53 | 0.04 | 0.40 |
| ePt_571164 | LG11 | 72.78 | 0.07 | 0.09 | ePt_504458 | LG10 | 28.94 | 0.07 | 0.20 |
| ePt_503841 | LG11 | 87.60 | 0.24 | 0.05 | ePt_643963 | LG10 | 36.64 | 0.09 | 0.24 |
|  |  |  |  |  | ePt_568851 | LG10 | 42.77 | 0.11 | 0.23 |
|  |  |  |  |  | ePt_644176 | LG10 | 45.86 | 0.05 | 0.17 |
|  |  |  |  |  | ePt_600200 | LG10 | 50.88 | -0.03 | 0.57 |
|  |  |  |  |  | ePt_566772 | LG10 | 62.32 | -0.11 | 0.64 |
|  |  |  |  |  | ePt_638929 | LG10 | 66.87 | -0.11 | 0.62 |
|  |  |  |  |  | ePt_574725 | LG10 | 77.32 | -0.04 | 0.16 |
|  |  |  |  |  | ePt_575312 | LG10 | 87.79 | -0.13 | 0.23 |
|  |  |  |  |  | ePt_640871 | LG10 | 91.92 | -0.18 | 0.28 |
|  |  |  |  |  | ePt_571374 | LG11 | 0.00 | 0.11 | 0.07 |
|  |  |  |  |  | ePt_638531 | LG11 | 2.23 | 0.08 | 0.11 |
|  |  |  |  |  | ePt_567666 | LG11 | 11.06 | 0.13 | 0.14 |
|  |  |  |  |  | ePt_575643 | LG11 | 25.68 | 0.01 | 0.27 |
|  |  |  |  |  | ePt_504138 | LG11 | 29.34 | -0.02 | 0.26 |
|  |  |  |  |  | ePt_504803 | LG11 | 46.11 | -0.07 | 0.15 |
|  |  |  |  |  | ePt_570513 | LG11 | 52.30 | -0.09 | 0.14 |
|  |  |  |  |  | ePt_600156 | LG11 | 60.11 | -0.11 | 0.10 |
|  |  |  |  |  | ePt_573069 | LG11 | 69.61 | -0.10 | 0.07 |
|  |  |  |  |  | ePt_503383 | LG11 | 90.66 | 0.07 | 0.11 |

SD, Standard deviation. QTL effects at marker positions based on composite interval mapping (Model 6, QTL Cartographer v2.5).

^a^ Marker data in the F1 hybrid maps were recoded so that positive and negative additive effect values on all linkage groups are associated with the effect of replacement of the *E. grandis* allele with the *E. urophylla* allele in backcross progeny. Linkage phases were arbitrary from one linkage group to the next for the backcross parents and the directions of the effect are therefore not indicated for these parents.
